# Dual Feedforward Loops Modulate Type I Interferon Responses and Induce Selective Gene Expression during TLR4 Activation

**DOI:** 10.1016/j.isci.2020.100881

**Published:** 2020-02-01

**Authors:** Jie Zhou, Tingzhe Sun, Shouheng Jin, Zhiyong Guo, Jun Cui

**Affiliations:** 1 MOE Key Laboratory of Gene Function and Regulation, State Key Laboratory of Biocontrol, School of Life Sciences, Sun Yat-sen University, Guangzhou, Guangdong 510006, China; 2 School of Life Sciences, Anqing Normal University, Anqing, Anhui 246011, China; 3 Organ Transplant Center, The First Affiliated Hospital, Sun Yat-sen University, Guangzhou 510080, China

**Keywords:** Biological Sciences, Cell Biology, Integrative Aspects of Cell Biology, Mathematical Biosciences

## Abstract

Although the dynamic features of type I coherent feedforward loop (C1-FFL) has been well studied, how C1-FFL shapes cell-to-cell heterogeneity remains unclear. Here, we found that C1-FFL with OR logic serves as “noise reducer,” whereas C1-FFL with AND logic functions as “noise propagator” to fine-tune the heterogeneity of signaling molecule's activation. Within Toll-like receptor 4 (TLR4) signaling pathway, we demonstrated that MyD88 together with TRIF generates a C1-FFL to control TBK1 phosphorylation and reduce its cell-to-cell heterogeneity, whereas noisy TRIF activation induced high heterogeneity of IRF3 activation through another C1-FFL. We further developed a mathematical model with dual C1-FFLs to uncover how MyD88 and TRIF encoded differential dynamics for TBK1 and IRF3 activation. Integration of dual FFLs drives MyD88-TBK1 axis to determine the specificity of IFN-stimulated genes transcription. Collectively, our work elucidates a paradigm that tunable TLR4-mediated type I IFN responses are subtly controlled by dual FFLs.

## Highlights

• MyD88 directly activates TBK1 under LPS stimulation
• MyD88 and TRIF form a dual FFL to regulate heterogeneity of TBK1 and IRF3
• FFLs with different logic show opposite effect on heterogeneity transduction
• MyD88-TBK1 axis determines the specificity of the downstream gene transcription

## Graphical Abstract

**Figure:**
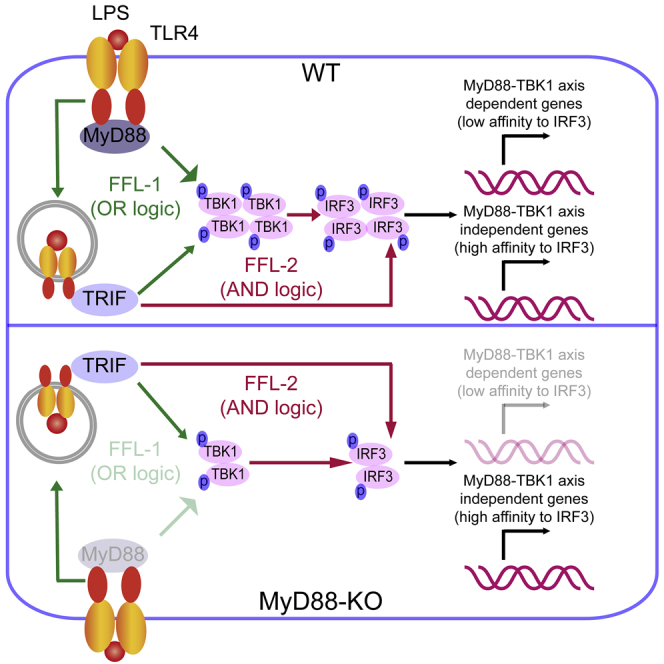

## Introduction

Signaling networks describe the interaction among signaling molecules, including receptors, adaptors, and kinases and so on (Alon, 2007). Within signaling networks, various kinds of network motifs control the signal transduction and provide specific characteristics for the network output. For instance, positive feedback loop can capture the slight input and amplify the signal to raise a high level of output (Slepchenko and Terasaki, 2004). Negative feedback loop can inhibit the signal transduction according to the level of input, which avoids a harmful, excessive output and turns the system back to equilibrium (Tyson et al., 2003). Moreover, combination of diverse network motifs results in certain special characteristics, such as bistability and so-called switch-like behavior raised by double-negative feedback loops (Ferrell, 2002).

Feedforward loop (FFL), consisting of at least three molecules and three regulatory reactions, is one of the most important network motifs that appears in hundreds of signaling networks. According to the regulatory reactions (positive or negative), eight types of FFL are defined among which type I coherent FFL (C1-FFL) is the common type of FFL (Alon, 2007). Within C1-FFL, the logic gate controls the exact way A and B activate C. AND logic means that activation of C requires both A and B, whereas OR logic indicates that either A or B is enough for C activation. Different kind of logics provide totally different characters for C1-FFL. C1-FFL with AND logic is thought as a “sign-sensitive delay” that it introduces a delay for output C (Kalir et al., 2005). Otherwise, C1-FFL with OR logic allows the continued production of output facing a transient loss of input (Mangan et al., 2006). Accumulating evidence showed that cell-to-cell heterogeneity of transcriptional factor activation plays an important role in the regulation of cell signaling (Gordon and Taylor, 2005, Hughey et al., 2015). However, the characters of C1-FFL in cell-to-cell heterogeneity processing remains largely unclear.

Innate immunity, as the first line of defense against invading pathogens, is equipped with a large group of pattern recognition receptors (PRRs) like Toll-like receptors (TLRs). TLR4 recognizes lipopolysaccharide (LPS) from the outer cell membrane of Gram-negative bacteria (Poltorak et al., 1998, Shibata et al., 2011) and transduces signals through both myeloid differentiation marker 88 (MyD88) and TIR domain-containing adaptor protein-inducing interferon-β (TRIF) as its adaptors to induce innate immune responses (Kawai and Akira, 2010). The whole scheme of TLR4 signaling pathway has been well studied previously (Garantziotis et al., 2008, West et al., 2006). TLR4 could recognize LPS and recruits MyD88 at plasma membrane and induces the oligomerization of MyD88 to form Myddosome (Lin et al., 2010). Myddosome activates inhibitor of κB (IκB) kinase (IKK) complex, resulting in the phosphorylation and degradation of IκB and the activation of nuclear factor κB (NF-κB) (Kawai and Akira, 2010). Furthermore, activated TLR4 undergoes endocytosis and endosome translocation to interact with TRIF (Tanimura et al., 2008). TRIF is reported to activate IRF3 in two steps, activating TBK1 through TRAF3 (Hacker et al., 2006) and recruiting IRF3 through TRIF phosphorylation by TBK1 (Liu et al., 2015), thus leading to the nuclear translocation of IRF3, as well as the expression of interferon-β (IFN-β) and hundreds of downstream IFN-stimulated genes (ISGs) (Oganesyan et al., 2006).

TLR4-induced cell-to-cell heterogeneity of NF-κB activation at single-cell level has been well studied: oscillatory activation of NF-κB and temporal pattern of downstream gene induction are controlled by negative feedback of IκB family (Hoffmann et al., 2002, Nelson et al., 2004), whereas dynamic features of NF-κB activation is encoded by MyD88 and TRIF (Cheng et al., 2015). However, how the single-cell behavior, like cell-to-cell heterogeneity, of IRF3 activation is controlled within the TLR4 signaling pathway remains unknown.

Here, we first uncovered that C1-FFL is significant for cell-to-cell heterogeneity regulation, whereby C1-FFL serves as “noise propagator” with AND logic and “noise reducer” with OR logic, respectively. Within the TLR4 signaling pathway, we found that MyD88, a well-known IKK activator, could also strongly enhance TBK1 phosphorylation in a TRIF-independent manner, forming a C1-FFL with OR logic with TRIF to shape TBK1 activation. Liu et al. reported that both TRIF and TBK1 were necessary for IRF3 activation (Liu et al., 2015), indicating another C1-FFL with AND logic involved in IRF3 activation. Thus, we constructed an optimized dual feedforward loop model for TLR4-induced IRF3 activation, in which C1-FFL precisely controlled the cell-to-cell heterogeneity of activation of TBK1 and IRF3, and found that these FFLs also contribute largely to the selective expression of downstream ISG genes by manipulating the level and cell-to-cell heterogeneity of TBK1 and IRF3 activation. Finally, our findings provided critical insight into how C1-FFLs modulate the activation of signaling proteins and determine the specificity of the downstream gene expression.

## Results

### Diverse Cell-to-Cell Heterogenetic Patterns of the IRF3 Nuclear Translocation and TBK1 Activation in TLR4 Signaling

**Figure 1:**
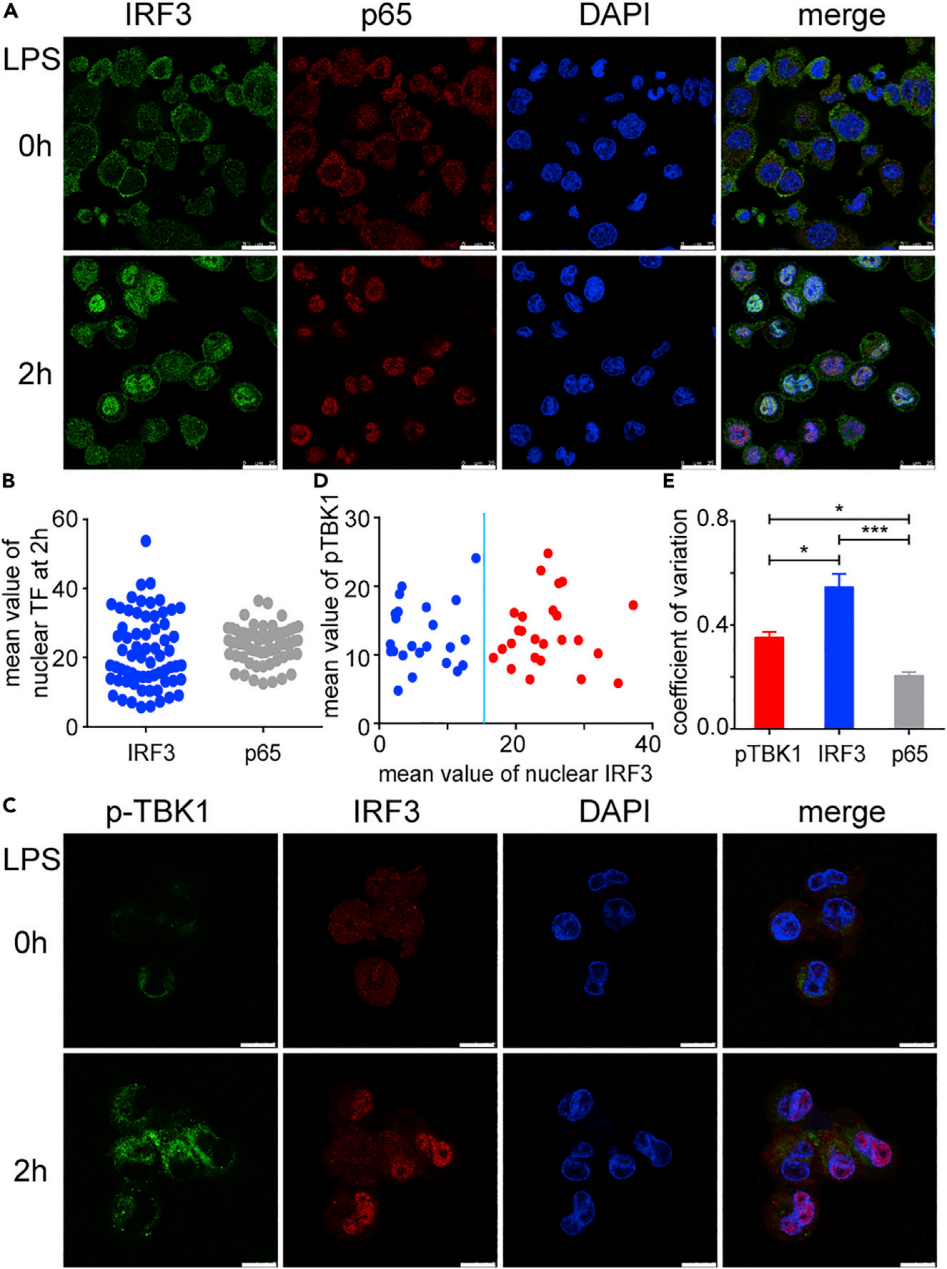
Diverse Patterns of Nucleus Translocation of IRF3 and p65 Were Observed under LPS Stimulation in THP-1-Derived Macrophages (A) Wild-type (WT) THP-1-derived macrophages were treated with LPS (200 ng/mL) for indicated time points, and nuclear translocation of IRF3 and p65 were imaged by con-focal microscope. (B) The relative mean value of nuclear IRF3 and p65 were measured by ImageJ. (C) TBK1 phosphorylation together with IRF3 nuclear translocation was also captured by confocal microscope. (D) The relative mean value of phosphorylated TBK1 and nuclear IRF3 in single cells (n = 48) of (D) were measured by ImageJ. Cells with different level of nuclear translocated IRF3 were divided into two groups (blue group with lower level of IRF3 nuclear translocation and red group with higher level of IRF3 nuclear translocation). (E) Coefficient of variation of TBK1 phosphorylation and nuclear translocation of IRF3 and p65 were calculated. More than 48 cells were analyzed for each group. Data are representative of three independent experiments (shown as mean ± SEM in [C]). * p < 0.05, *** p < 0.001. The magnification in (A) is ×400, and the scale bar represents 25 μm, whereas in (D) the magnification is ×1,000, and the scale bar represents 10 μm. See also Figure S1.

The TLR4 signaling pathway is one of the most well-known innate immune pathways, in which MyD88 and TRIF forms C1-FFLs to modulate the activation of both NF-κB and IRF3 for downstream genes induction. On the one hand, temporal profiles of NF-κB activation under LPS stimulation were thought to be shaped by a C1-FFL with OR logic (Hoffmann et al., 2002, Kearns et al., 2006, Nelson et al., 2004, Werner et al., 2005). On the other hand, Liu et al. described that both TRIF and TBK1 controlled IRF3 activation in the TLR4 signaling pathway (Liu et al., 2015). In order to figure out how C1-FFL regulates heterogeneity of the TLR4 signaling pathway, we first detected the nuclear translocation of IRF3 and p65 (a major NF-κB subunit within TLR4 signaling) by immunofluorescence (IF) assay in THP-1-derived macrophages under LPS stimulation (Figure 1A). As expected, the nuclear translocation of IRF3 showed obvious cell-to-cell variability compared with the nuclear translocation of p65 (Figures 1B and 1C). We wondered whether the phosphorylation of TBK1, which is upstream of IRF3 activation, could display a similar activation pattern. Surprisingly, IF experiments and further quantitation showed that the phosphorylation level of TBK1 was less varied among cell population, whereas heterogeneity of IRF3 nuclear translocation was higher, which allowed easy division of cells into two groups based on the level of IRF3 nuclear translocation but not phosphorylation level of TBK1 (Figure 1D). We have further compared the coefficient of variation in our data of TBK1 phosphorylation and nuclear translocation of IRF3 and p65 with statistical tests and found significant difference between IRF3 and TBK1 or between IRF3 and p65 (Figure 1E), indicating the possibility that TBK1 activation was controlled by unknown mechanism that regulated its heterogeneity.

Previous study indicated that noise from TLR4 endocytosis-dependent TRIF activity was more important for variation in NF-κB dynamics than that from MyD88 pathway (Cheng et al., 2015). To validate that TRIF was the source of cell-to-cell heterogeneity of IRF3 activation, we treated cells with poly (I:C), which specifically activates TRIF through TLR3 (Bell et al., 2006), and found that the phosphorylation of TBK1 and the nuclear translocation of IRF3 displayed strong correlation in THP-1-derived macrophages (Figures S1A–S1C). We also found similar pattern of TBK1 phosphorylation and IRF3 nuclear translocation in MyD88-KO THP-1-derived macrophages with comparable level of phosphorylated TBK1 in both cell types (Figures S1D–S1F), implying that TRIF serves as a heterogeneity introducer for both TBK1 and IRF3 in TLR3 pathway.

### IRF3 Nuclear Translocation Requires Both MyD88 and TRIF upon TLR4 Activation

**Figure 2:**
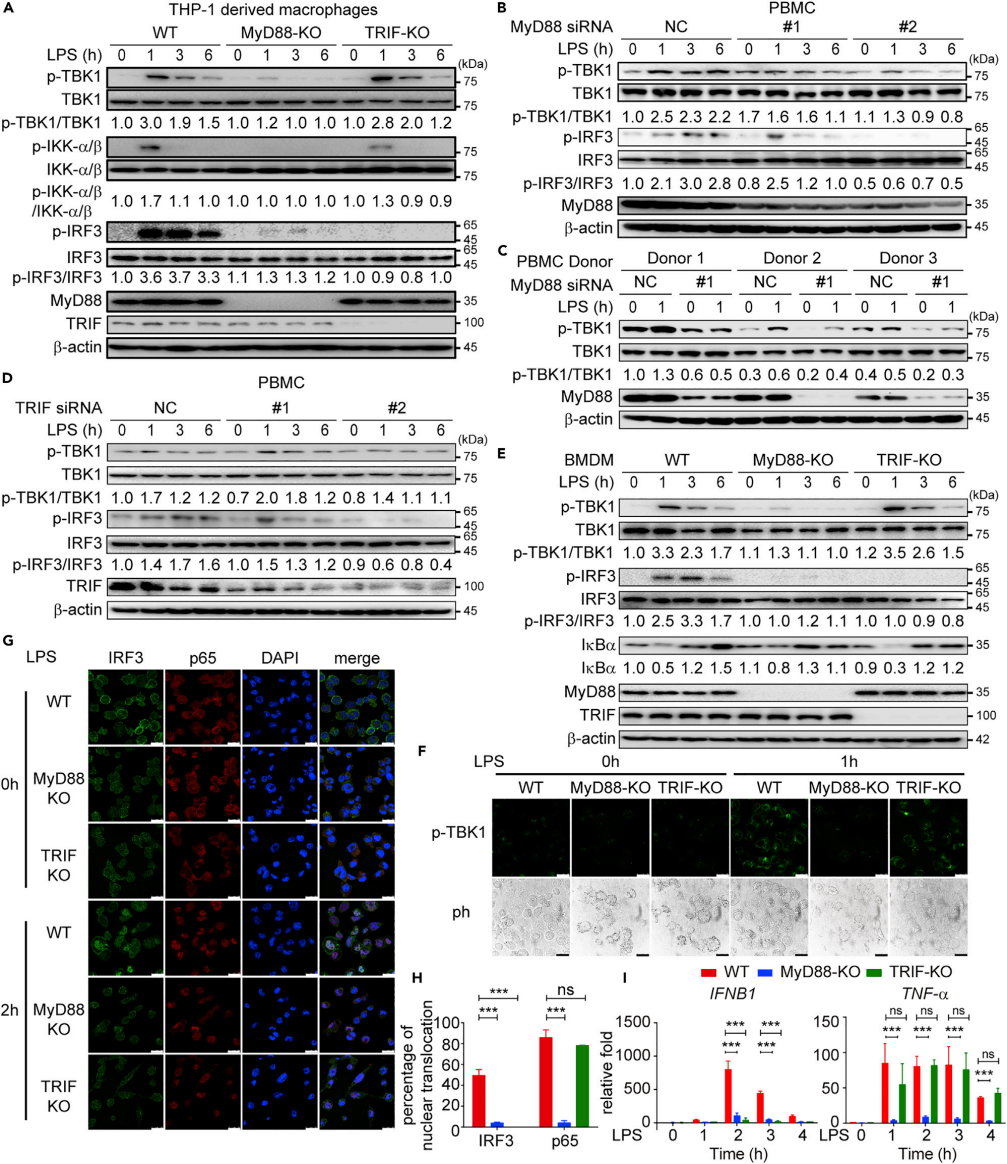
Both MyD88 and TRIF Are Critical for IRF3 Activation by LPS Treatment (A and B) Wild-type (WT), MyD88-knockout (KO), and TRIF-KO THP-1-derived macrophages (A) and peripheral blood mononuclear cells (PBMCs) transfected with or without *MyD88* siRNA (B) were stimulated with LPS (200 ng/mL) for indicated time points. Phosphorylation of indicated proteins was detected by immunoblot (IB) analyses. (C) PBMCs from three different donors with or without *MyD88* siRNA transfection were stimulated with 200 ng/mL LPS for 1 h or left untreated. Phosphorylation of TBK1 was detected by IB analysis. (D) PBMCs transfected with or without *TRIF* siRNA were stimulated with LPS (200 ng/mL) for indicated time points. Phosphorylation of indicated proteins was detected by IB analyses. (E) Wild-type (WT), MyD88-knockout (KO), and TRIF-KO bone marrow-derived macrophages (BMDMs) were stimulated with LPS (200 ng/mL) for indicated time points. Protein level of indicated proteins was detected by IB analyses. (F–H) Phosphorylation of TBK1 (F) as well as nuclear translocation of IRF3 and p65 (G) was imaged by confocal microscope and quantified by ImageJ (H); 50 cells were analyzed for each group. (I) Expression of IFN-β and TNF-α was detected by quantitative real-time PCR (qRT-PCR). Data of (A)–(G) are representative of three independent biological experiments; data are shown as mean ± SEM of three independent biological experiments in (H) and (I). ns, not significant (p > 0.05); *** p < 0.001 The magnification of the images is ×400, and the scale bar represents 25 μm. See also Figure S2.

To investigate whether MyD88 restricts the heterogeneity of TBK1 activation, we constructed MyD88-KO and TRIF-KO THP-1 cells using CRISPR-Cas9 system (Figure S2) and tested the features of TBK1 and IRF3 activation. As expected, we found that phosphorylation of IKK-α/β was abolished in MyD88-KO cells but not TRIF-KO cells under LPS stimulation (Figure 2A). Unexpectedly, we observed that TBK1 phosphorylation was primarily dependent on MyD88 but not TRIF, whereas both MyD88 and TRIF were required for IRF3 activation by LPS treatment (Figure 2A). To exclude the possibility that transformed cells had signaling characteristics distinct from primary immune cells, we further detected the phosphorylation of TBK1 in human peripheral blood mononuclear cells (PBMCs) with or without MyD88 knockdown (MyD88-KD). Significant decrease of TBK1 phosphorylation was observed when MyD88 expression was silenced (Figures 2B and 2C), whereas decrease of IRF3 phosphorylation but not TBK1 phosphorylation was observed in TRIF-KD PBMCs (Figure 2D). In addition, similar effect of the activation of TBK1 and IRF3 was observed in MyD88-KO or TRIF-KO bone marrow-derived macrophages (BMDMs) (Figure 2E). We next detected the phosphorylation of TBK1 (Figure 2F) and nuclear translocation of IRF3 and p65 (Figures 2G and 2H) through IF assay, which further supported our conclusion that MyD88 is required for TBK1 activation under LPS treatment. We also confirmed that both MyD88 and TRIF were required for the induction of IFN-β by LPS treatment, whereas MyD88 but not TRIF mainly controlled the expression of pro-inflammatory cytokines, such as TNF-α (Figure 2I). Collectively, our results suggested that both MyD88 and TRIF affected IRF3 activation in TLR4 signaling, whereas MyD88 plays a dominant role in TBK1 phosphorylation.

### MyD88 Interacts with TBK1 to Induce Its Oligomerization and Phosphorylation

**Figure 3:**
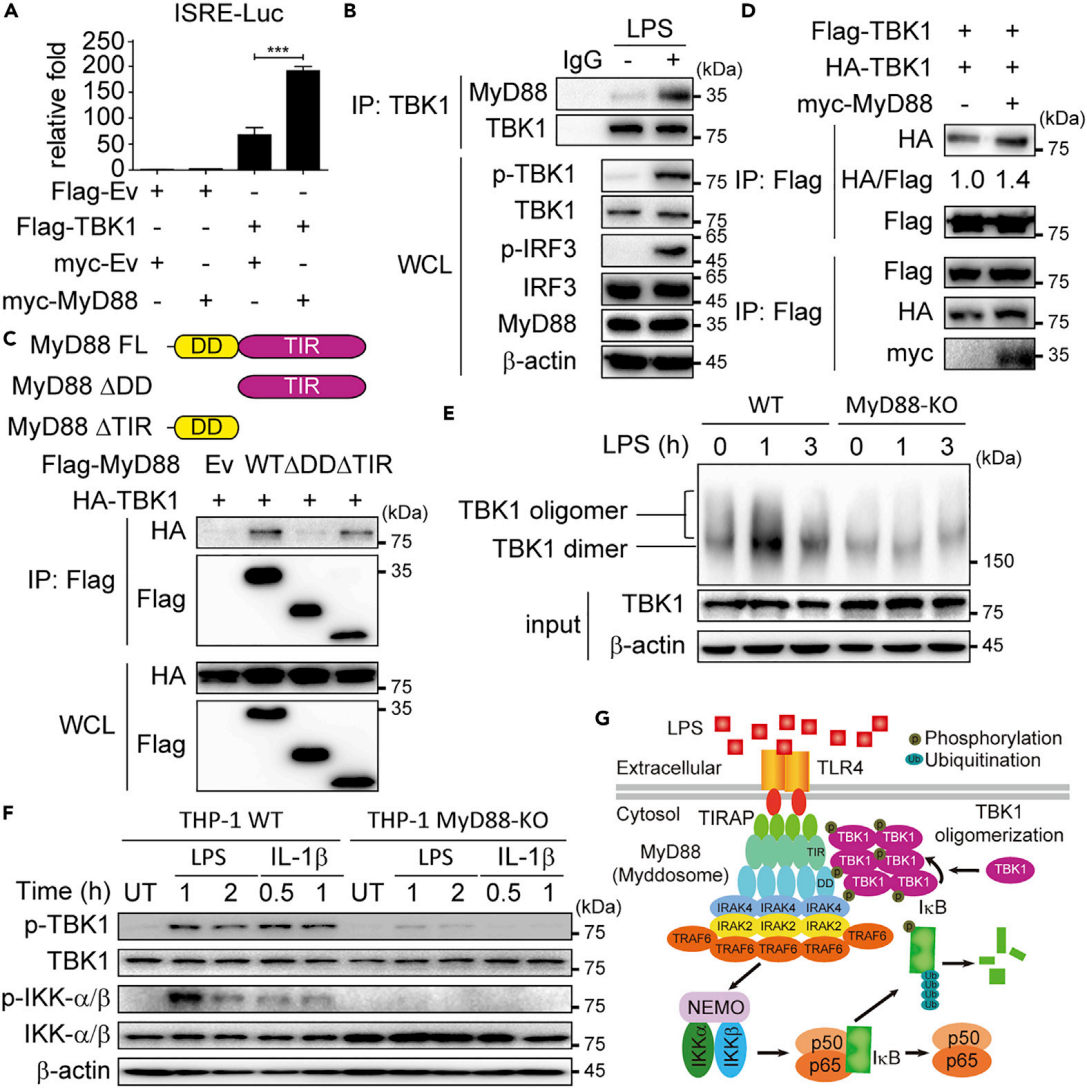
MyD88 Activates TBK1 through TIR Domain (A) HEK 293T cells were transfected with plasmids encoding ISRE luciferase reporter and Flag-TBK1, together with expression vector for myc-MyD88 or empty vector (Ev). (B) Co-immunoprecipitation (IP) and immunoblot (IB) analyses were performed for THP-1-derived macrophages stimulated with LPS (200 ng/mL) for 1 h with indicated antibodies. (C) HEK 293T cells transfected with HA-TBK1 and its deletion mutants. Whole-cell lysates (WCL) were immunoprecipitated with anti-Flag beads, followed by IB analyses with anti-HA antibody. HEK 293T cells were transfected with Flag-TBK1 and HA-TBK1, together with empty vector and myc-MyD88. (D) Cell lysates were collected for IP with anti-Flag beads followed by IB analyses with anti-HA antibody. (E) Native gel electrophoresis assay of TBK1 oligomerization in wild-type (WT) and MyD88-knockout (KO) THP-1-derived macrophages under LPS stimulation (200 ng/mL) for indicated time points. (F) IB analysis of total and phosphorylated TBK1 and IKK-α/β in WT and MyD88-KO THP-1-derived macrophages stimulated with LPS (200 ng/mL) or IL-1β (10 ng/mL) or left untreated (UT). (G) Proposed model illustrating how TBK1 is activated by MyD88 under LPS stimulation. Data are representative of three independent biological experiments (shown as mean ± SEM in [A]), *** p < 0.001.

To uncover the molecular mechanisms by which MyD88 induced TBK1 phosphorylation, we first used an IFN-stimulated response element (ISRE) luciferase reporter (which requires IRF3 activity only) to confirm whether MyD88 enhanced the activation of IRF3 through TBK1. We found that overexpression of MyD88 alone failed to increase the activation of IRF3. However, IRF3 activation was markedly enhanced by MyD88 when TBK1 was co-overexpressed, indicating that augmentation of IRF3 activation by MyD88 relied on TBK1 (Figure 3A). Next, we wondered whether MyD88 interacted with TBK1 under LPS stimulation. We stimulated cells with LPS and harvested cell lysates at indicated time points. Enhanced interaction between TBK1 and MyD88 can be observed upon LPS treatment, indicating that TBK1 could be recruited by MyD88 in TLR4-mediated signaling (Figure 3B). To identify the domain of MyD88 responsible for TBK1 recruitment, we generated two deletion mutants of MyD88 containing the N-terminal DD domain or the C-terminal TIR domain, respectively. TIR domain of MyD88 could interact with TBK1, whereas DD domain failed to do so (Figure 3C). TBK1 was found to undergo oligomerization and trans-autophosphorylation after recruitment by certain adaptors (Ma et al., 2012). We observed that the oligomerization of TBK1 could be enhanced by overexpression of MyD88 (Figure 3D). Furthermore, oligomerization of TBK1 was significantly decreased in MyD88-KO THP-1-derived macrophages compared with wild-type (WT) macrophages under LPS stimulation (Figure 3E). Taken together, these data showed that MyD88 activated TBK1 through recruiting TBK1, inducing TBK1 oligomerization and phosphorylation. We next wonder whether other stimuli (e.g., interleukin-1β, IL-1β) that specifically activate MyD88 could also activate TBK1 through MyD88. Immunoblot assay showed that IL-1β was able to activate TBK1 in WT cells but not in MyD88-KO cells (Figure 3F), further validating that MyD88 is required to activate TBK1. Altogether, we provided a novel model for TBK1 activation mediated by MyD88. After LPS stimulation, MyD88 is recruited to TLR4 and formed myddosome. Then, MyD88 further recruits TBK1 through its TIR domain, leading to TBK1 oligomerization and autophosphorylation (Figure 3G).

### Cross Talk of MyD88 and TRIF Forms Dual Feedforward Loops and Modulates TBK1 and IRF3 Activation in TLR4 Signaling

Together with the well-known TRIF-TBK1 axis, the newly discovered MyD88-TBK1 axis generates a novel C1-FFL with OR logic in the TLR4 signaling network, which may effectively filter the heterogeneity for TBK1 activation introduced by TRIF. We next provided an optimized mathematical model to describe LPS-mediated TBK1/IRF3 activation with dual C1-FFLs (Figure S3A). We fitted the parameters based on previously reported models and our experimental data (Figure S3B). A local sensitivity analysis was performed for all kinetic parameters and initial conditions (Figure S3C). We found that parameters associated with MyD88 showed greater effect on TBK1 activation than those associated with TRIF, whereas all these parameters affected IRF3 activation, which is consistent with our experimental results.

Compared with IRF3 activation, NF-κB activation primarily depends on MyD88 (Kawai et al., 1999). Simulation of dose-response curve for TBK1/IRF3 activation showed that NF-κB activation was more sensitive to stimuli than IRF3 activation (Figure S3D), implying higher correlation between MyD88 and signal sensitivity. The calculation of the concentration for 50% of maximal effect (EC_50_) and Hill coefficients of IKK-α/β, TBK1, and IRF3 also indicated that MyD88-mediated activations of IKK-α/β and TBK1 were much more sensitive to the stimulation of LPS than that of IRF3 (Figure S3E).Taken together, IF assay showed higher signal sensitivity of NF-κB than IRF3, consistent with our simulation results (Figures S3F and S3G), which further confirmed the reliability of our model.

### Type I Coherent Feedforward Loop Shapes Heterogeneity Transduction of Signaling Molecule Activation

To figure out how C1-FFL affects the cell-to-cell heterogeneity of signaling molecule activation, we first compared different efficiency of heterogeneity transduction from a stochastic input by linear motif or C1-FFL with AND logic or OR logic for C activation (Figure S4A). When facing stochastic input, we found that C1-FFL with AND logic showed significantly higher efficiency for heterogeneity transduction of signaling molecule activation (B to C), whereas C1-FFL with OR logic and LINEAR motif failed to do so (Figure S4B). Since C1-FFL with AND logic needs the activity of both A and B to activate C, we wondered whether the strength of C activation by A and B, defined as k_3_ in our model, affects the heterogeneity transduction of signaling molecule activation. We found that efficiency of heterogeneity transduction of signaling molecule activation was reduced when the level of k_3_ was increased (Figure S4C). When the input of C1-FFL contains low variance, the reaction within C1-FFL may also introduce cell-to-cell heterogeneity, like endocytosis (Tanimura et al., 2008). Thus, the ability of C1-FFL to filter the heterogeneity is important to maintain the signal stability. We simulated C activation with stochastic activation of B and found that C1-FFL with OR logic can efficiently reduce the cell-to-cell heterogeneity compared with C1-FFL with AND logic (Figures S4D and S4E). We further discovered that the strength of C activation by A or B (defined as k4 and k3 in our model for C1-FFL with OR logic) and the ratio between them controlled the efficiency of heterogeneity transduction of signaling molecule activation. As the level of k_3_ and k_3_/k_4_ was increased, the ability of heterogeneity filter was enhanced in C1-FFL with OR logic (Figure S4F). Altogether, we concluded that C1-FFL played opposite roles in transduction of cell-to-cell heterogeneity of signaling molecule activation: “noise propagator” with AND logic and noise reducer with OR logic.

### Cell-to-Cell Heterogeneity of TBK1 and IRF3 Was Shaped by Dual Feedforward Loops in TLR4 Signaling Pathway

**Figure 4:**
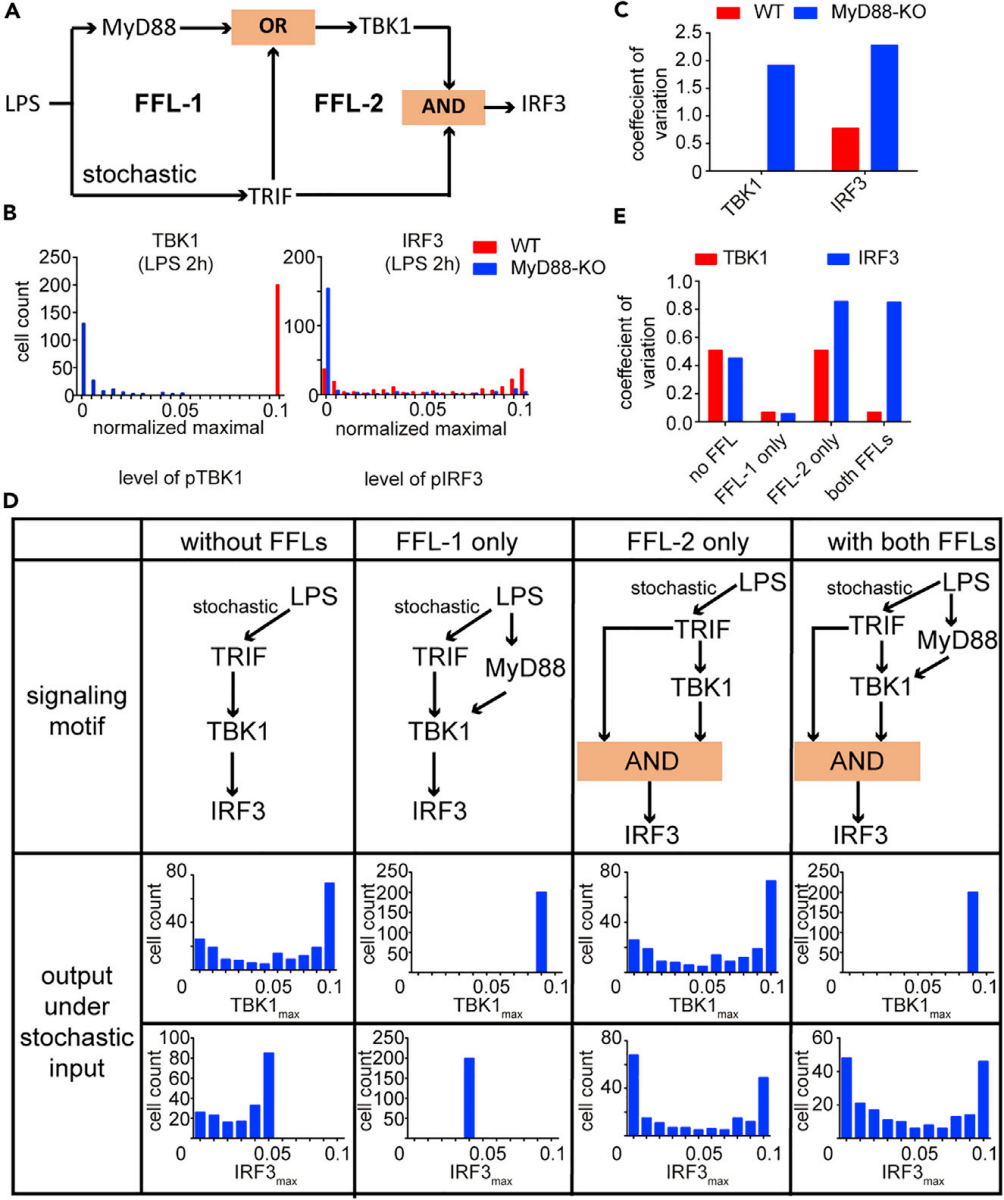
Dual C1-FFLs Shape the Heterogeneity of the Activities of Key Molecules in TLR4 Signaling Pathway (A) Schematic representation of feedforward loops (FLLs) within TLR4 signaling pathway; whole pathway of TLR4 signaling was shown in Figure S4A. (B and C) Maximal levels of activated TBK1 and IRF3 under LPS stimulation were simulated with or without MyD88 *in silico* (B), and coefficients of variation (CV) of TBK1 and IRF3 activation were calculated (C). (D and E) Maximal levels and coefficient of variation of TBK1 and IRF3 were compared under stochastic activation of TRIF in models with or without FFL-1 and FFL-2. See also Figure S4 and Tables S7 and S8.

Within the TLR4 signaling pathway, a novel C1-FFL with OR logic controlled TBK1 activation (Figure 4A, FFL-1), whereas IRF3 activation was governed by another FFL with AND logic (Figure 4A, FFL-2). We next evaluated the heterogeneity of TBK1 and IRF3 activation in our model via simulating with log-normal distributed stochastic k_4_ (activation parameter of TRIF by TLR4) (Figures 4B and 4C), and the result was similar to our IF assay data (Figure 1D) that activation of TBK1 showed a much lower level of cell-to-cell heterogeneity than that of IRF3 in TLR4 pathway. To figure out how FFLs with different logic gates affect the heterogeneity of TBK1 and IRF3 activation, we simulated them in simplified models with altered FFLs and then fitted the model with experimental data under stochastic TRIF activation (Figures 4D and 4E). In linear model without FFLs, phosphorylation of both TBK1 and IRF3 showed considerable cell-to-cell variability and heterogeneity, which are decreased as the signal passed down via the TRIF-TBK1-IRF3 axis (Figure 4D, first column). Decreased variations in phosphorylated TBK1 and IRF3 could be observed when only FFL-1 was considered (Figure 4D, second column), whereas heterogeneous IRF3 activation could be detected only with FFL-2 (Figure 4D, third column). In addition, cell-to-cell variability of TBK1 activation was restricted at markedly low level in stark contrast to that of IRF3 at the presence of both FFLs (Figure 4D, last column).

As we showed above, the ability of heterogeneity filter was controlled by the strength of C activation by A or B (Figure S4F). To figure out whether the value of k_3_ and k_4_, which denoted the strength of MyD88-induced and TRIF-induced TBK1 activation, affected coefficient of variation of TBK1 activation (CV_TBK1_), we calculated CV_TBK1_ with a series of k_3_/k_4_ using different fixed value of k_4_ and found that heterogeneity of TBK1 phosphorylation was restricted by the strength of MyD88-induced and TRIF-induced TBK1 activation (Figure S4G). Similar to the simulation above (Figure S4), our model showed that MyD88/TRIF-controlled FFL-1 could reduce cell-to-cell heterogeneity of TBK1, whereas FFL-2 was critical to effectively transduce cell-to-cell heterogeneity to IRF3, which provided efficient heterogeneity regulation within TLR4 signaling.

### MyD88-TBK1 Axis Is Indispensable for Selective Gene Expression

**Figure 5:**
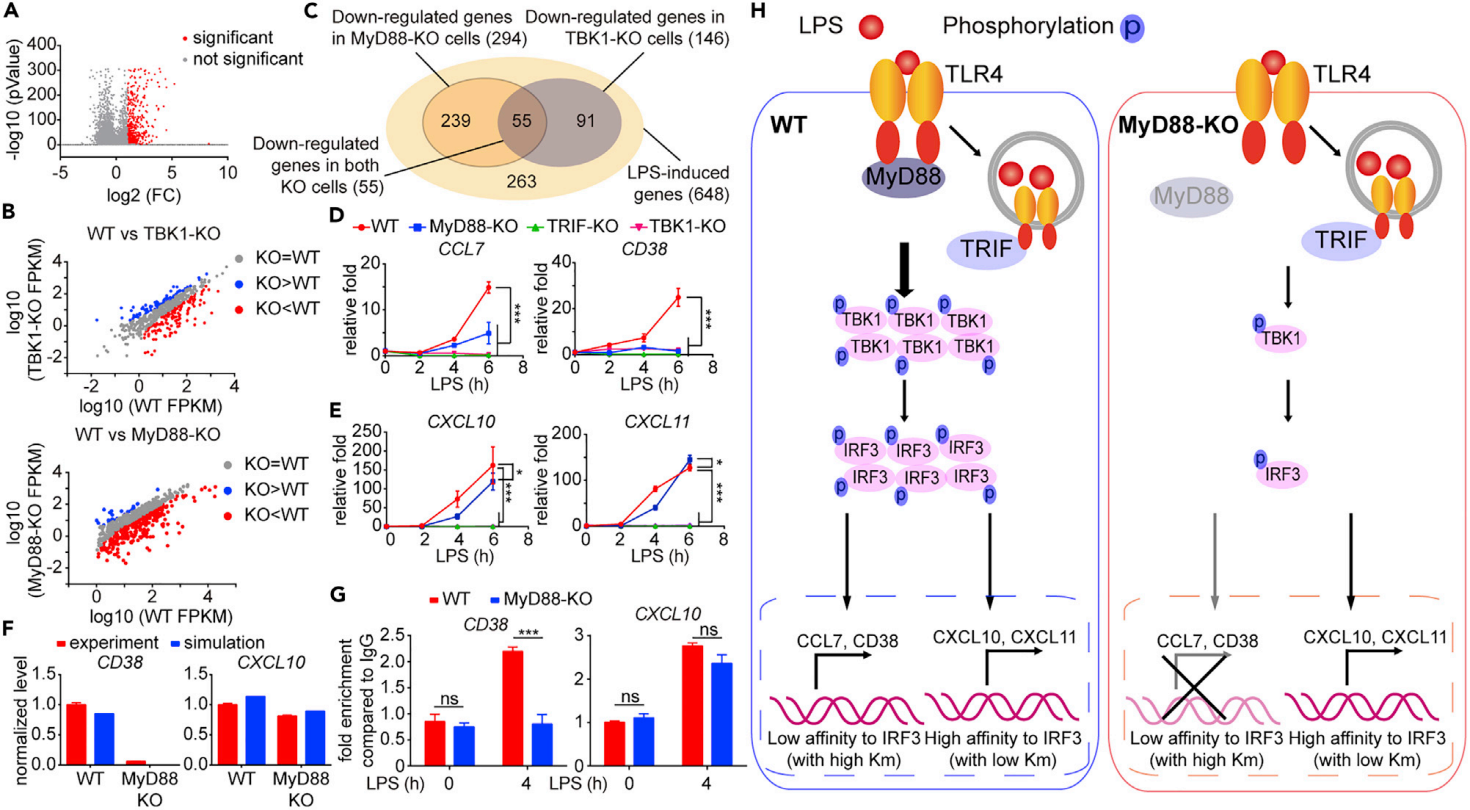
MyD88-TBK1 Axis Controls the Specificity of ISGs Transcription under LPS Stimulation (A) The up-regulated genes under LPS stimulation were marked with red points. The expression with at least two-fold changes and p < 0.05 was chosen (LPS treated versus untreated). (B) Pairwise comparison of FPKM of LPS-induced genes in wild-type (WT), MyD88-knockout (KO), and TBK1-KO THP-1-derived macrophages. Red points indicated that genes expression was two-fold down-regulated in KO cells, whereas blue points marked the up-regulated genes, among which p < 0.05. (C) Schematic of consistently down-regulated genes between MyD88-KO and TBK1-KO THP-1-derived macrophages. (D) Gene ontology analysis for down-regulated genes in both MyD88-KO and TBK1-KO cells. (E and F) quantitative real-time PCR (qRT-PCR) analysis of indicated genes in WT and other indicated KO THP-1-derived macrophages was performed under LPS stimulation for 6 h. (G) ChIP-qPCR assay for IRF3 at CD38 or CXCL10 gene promotor region in WT and MyD88-KO THP-1-derived macrophages stimulated with LPS for 4 h or left untreated. (H) Comparison of induction of CD38 and CXCL10 between experimental data and simulation data under LPS stimulation for 4 h. Proposed model illustrating how MyD88-induced TBK1 activation controls the induction of genes, like CCL7 and CD38 (H). Data in (E)–(G) are plotted as mean ± SEM of three independent biological experiments. ns, not significant (p > 0.05); * p < 0.05, ***p < 0.001. See also Figure S5.

Since TBK1 mainly serves as a kinase of IRF3 after LPS stimulation, it seems perplexing that TBK1 and IRF3 showed different levels of heterogeneity, which might be a waste of energy for TBK1 activation in IRF3-inactivated cells. To address this question, we detected the phosphorylation level of IKK-α/β, MAPK p38, and JNK, which controlled the inflammatory responses under LPS stimulation. Diminished activation of IKK-α/β but not MAPK p38 and JNK was observed (Figure S5A), which was consistent with previous reports (Tojima et al., 2000), indicating that TBK1 enhanced the activation of NF-kB signaling in both IRF3-activated and IRF3-inactivated cells. Next, we wondered whether MyD88-TBK1 axis regulated downstream genes expression. We performed transcriptome sequencing to figure out the function of MyD88-TBK1 axis. Compared with untreated cells, the expression of 648 genes was induced by LPS stimulation (Figure 5A). We found that 294 genes were down-regulated when MyD88 was knocked out and expression of 146 genes were blocked in TBK1-KO cells (Figure 5B), among which 55 genes were found to be consistently down-regulated in both MyD88-KO and TBK1-KO cells (Figure 5C). In order to compare the novel role of MyD88-TBK1 axis to the established function of TRIF in the TLR4 signaling pathway, we detected the induction of overlapped genes (controlled by both MyD88 and TBK1) in TRIF-KO cells through qRT-PCR assays and found that expression of overlapped genes were abolished in TRIF-KO cells (Figure S5B). Taken together, Liu et al. reported that TRIF is necessary for recruitment of TBK1 and IRF3 and further activation of IRF3 (Liu et al., 2015). Therefore, we preliminarily concluded that the novel role of MyD88-TBK1 axis was dependent on TRIF-TBK1-IRF3 axis. We then performed gene ontology analysis using DAVID (Huang et al., 2009) and found that 55 genes regulated by both MyD88 and TBK1 were mainly associated with inflammation and chemotaxis (Figure S5C, upper panel). Next, we analyzed the pathway for genes that were down-regulated in MyD88-KO and TBK1-KO, respectively (Figure S5C, middle and lower panel). Compared with the pathway analysis of genes regulated by both MyD88 and TBK1, we found that pathway clusters of each groups were very similar, indicating that MyD88 and TBK1 contained similar functions in the TLR4 signaling pathway. With further quantitative real-time PCR (qRT-PCR) validation, we found that MyD88-TBK1 axis was indispensable for induction of certain ISGs, such as CCL7 and CD38 (Figure 5D), which cannot be activated by TRIF alone or TLR3 agonist (Figure S5D). However, the expression level of other ISGs, such as CXCL10 and CXCL11, were not significantly affected by MyD88-TBK1 axis at later time points (Figure 5E). To distinguish the induction of MyD88-dependent and independent genes in our mathematical model, different IRF3 affinities of promoters of MyD88-dependent and independent genes were hypothesized. MyD88-dependent genes contained lower IRF3 binding affinity because induction of these genes needed a higher level of IRF3 phosphorylation and nuclear translocation, whereas IRF3 binding affinities of MyD88-independent genes were higher. In our model, Hill kinetics (Hill coefficient = 1) was used to describe the process that activated IRF3 bound to the promoter of downstream genes and induced their expression, and diverse values of the dissociation constants were chosen to indicate the diverse IRF3 binding affinity. The distinct expression patterns of downstream genes were simulated by our mathematical model with different levels of binding affinity of IRF3 (Figure 5F). To prove our hypothesis, we compared the affinity of IRF3 binding to the promoters of CD38 (MyD88 dependent) and CXCL10 (MyD88 independent) in WT and MyD88-KO THP-1-derived macrophages under LPS stimulation with ChIP-qPCR assay. We found comparable binding of IRF3 on CXCL10 promoter in both WT and MyD88-KO cells under LPS stimulation, whereas that of CD38 showed significant difference between WT and KO cells (Figure 5G). Collectively, these results suggested that the MyD88-TBK1 axis served as a robust switch for IRF3 activation to overcome the threshold of diverse MyD88-dependent downstream genes and determined the selective gene expression (Figure 5H).

## Discussion

C1-FFL is one of the most important network motifs that regulate signaling transduction in bacteria (Shen-Orr et al., 2002), yeast (Lee et al., 2002), and other organisms (Odom et al., 2004), which is widely distributed among biochemical processes (Chang et al., 2014, Ryu et al., 2015). However, few reports focused on how C1-FFL regulated cell-to-cell heterogeneity of signaling molecule activation in signaling pathway. Here, our mathematical simulation uncovered that C1-FFL with AND logic maintained the heterogeneity of a stochastic input, whereas C1-FFL with OR logic could filter the heterogeneity of input, in which the strength of heterogeneity filter was affected by the ratio between the ability for A and B to activate output C. Cell-to-cell heterogeneity could result in activation in a portion of cells while the others remained inactivated. It was important for proper responses toward stimulation without excessive harmful output but did harm to the physiological processes that were necessary for the entirety of cell population. Thus, combination of C1-FFLs with AND logic and OR logic provided a solution to precisely control the cell-to-cell heterogeneity of different molecules.

TLR4 is the first discovered PRR in innate immune responses (Medzhitov et al., 1997). In response to Gram-negative bacteria, TLR4 activates both MyD88 and TRIF, leading to NF-κB and type I IFN responses (Lester and Li, 2014). However, how C1-FFL contributes to cell-to-cell heterogeneity of signaling molecule activation during TLR4 signaling remains largely elusive. In this study, diverse heterogenetic patterns of TBK1 and IRF3 activation were observed under LPS stimulation (through TLR4 pathway) but not poly (I:C) stimulation (through TLR3 pathway). MyD88 knockout cells also showed deficient activation of TBK1 and IRF3, indicating the functional relevance of MyD88 in TBK1 activation. Our experimental data further confirmed that TBK1 was mainly activated by MyD88 but not TRIF during LPS stimulation. The distinct pathways of TBK1 activation by MyD88 and TRIF formed a C1-FFL with OR logic to subtly control TBK1 activity and effectively reduced the heterogeneity level of TBK1 activation in TLR4 signaling.

Liu et al. reported that TRIF and TBK1 are indispensable for IRF3 activation (Liu et al., 2015), whereas other studies indicated that IL-1α might activate TBK1 using unknown kinases (Clark et al., 2009) that failed to activate IRF3 owing to the lack of phosphorylated TRIF (Liu et al., 2015). Here, we provided a mathematical model that incorporated dual FFLs for the TLR4 signaling pathway (Figure 3B) and showed that C1-FFLs with different logic gates differentially affect heterogeneity transduction of signaling molecule activation. Cheng et al. described that MyD88 and TRIF function as noise reducer and noise propagator, respectively, for NF-κB activation (Cheng et al., 2015). Here, our model pointed out that C1-FFLs with different logic did serve as the noise reducer and noise propagator within type I IFN responses. For TBK1 activation, C1-FFL with OR logic served as a noise reducer that restricted the level of variation of TBK1. Besides, C1-FFL with AND logic that controlled IRF3 phosphorylation could transduce cell-to-cell heterogeneity into activation of IRF3 directly from TRIF, acting as a noise propagator. Taken together, these chimeric FFLs in the TLR4 signaling pathway generate different levels of heterogeneity (i.e., low variation in MyD88-TBK1 axis and high variation in TRIF-IRF3 axis) to exert specific biological functions in innate immune responses.

TBK1 is reported to activate IRF3 and type I IFN in TLR-mediated signaling (Drexler and Foxwell, 2010, Kawai and Akira, 2010). MyD88-mediated TBK1 activation might provide a redundant way to activate IRF3 in combination with TRIF. We found that MyD88-TBK1 axis can enhance TBK1 and IRF3 activation, which could overcome the threshold of specific MyD88-TBK1-regulated genes, whereas TRIF could only activate markedly low levels of IRF3 to induce the expression of MyD88-independent genes. We found that expression of certain genes was dependent on the MyD88-TBK1 axis, which regulated innate immune responses for further pathogen clearance, such as CCL7 and CD38. CCL7 is the chemokine that recruits monocytes from bone marrow (Jia et al., 2008), and CD38 is a transmembrane protein that contains multiple functions involved in the regulation of innate systems (Quarona et al., 2013), such as uptake of *Listeria monocytogenes* by macrophages (Lischke et al., 2013). Therefore, the MyD88-TBK1 axis might specifically promote the inflammatory response by recruiting monocyte/macrophage and regulating the function of these cells. On the other hand, MyD88-independent genes may also play important roles in adaptive immunity, like CXCL10 and CXCL11. CXCL10 and CXCL11 are the ligands of CXCR3 that recruit T helper 1 cells and initiate adaptive immune responses (Lasagni et al., 2003). Taken together, the signaling network incorporated with dual C1-FFLs precisely balanced the innate and adaptive immune responses upon pathogen invasion by manipulating the cell-to-cell heterogeneity and activity of diverse signaling proteins.

Since the participation of MyD88 is one of the most important differences between TLR3 and TLR4 signaling pathways in the induction of type I IFN (Lester and Li, 2014), our findings of MyD88-TBK1 axis partially explain the ISG expression specificity between TLR3 and TLR4 signaling in response to diverse invading pathogens. However, IL-1β activates TBK1 through MyD88 but fails to activate IRF3 and induce expression of IFN and ISGs, leaving an open question that whether MyD88-activated TBK1 has other functions independent of IRF3 and IFN.

In summary, our findings have demonstrated a novel paradigm for dual C1-FFLs within the TLR4 signaling pathway to shape the dynamic responses in TBK1/IRF3 activation and provide specificity in gene expression profiles. By this means, our study may provide new insights for the complex regulation of cell responses through interaction among multiple signaling motifs rather than single molecule or signaling motif.

### Limitations of the Study

This study uncovered the novel role of how MyD88 controlled the activation of TBK1 within the TLR4 signaling pathway and provided an optimized model containing dual feedforward loops that precisely controlled the activation of TBK1 and IRF3, which further regulated the specific downstream genes expression profile. However, the function of MyD88-TBK1-regulated genes within immune systems, like effect of immune cells recruitment or activation of adaptive immunity, needs to be further investigated.

## Methods

All methods can be found in the accompanying Transparent Methods supplemental file.

## Data and Code Availability

All data needed to evaluate the conclusions in the paper are present in the paper and/or the Supplemental Materials. Additional data related to this paper may be requested from the authors.

## Supplemental Information

Document S1. Transparent Methods, Figures S1–S5, and Tables S1–S8

Data S1. Chimeric Feedforward Loops Models of TLR4 Signaling Pathway, Related to Figure 4
