# Dual Feedforward Loops Modulate Type I Interferon Responses and Induce Selective Gene Expression during TLR4 Activation

**DOI:** 10.1016/j.isci.2022.104748

**Published:** 2022-08-04

**Authors:** Jie Zhou, Tingzhe Sun, Shouheng Jin, Zhiyong Guo, Jun Cui

## Main text

(iScience *23*, 100881; February 21, 2020)

During preparation of the revised manuscript, the same images of p-TBK1 and p-IRF3 for Figures 2A and 2E and p-IRF3 for Figures 2B and 2D were used. Furthermore, the wrong image of IRF3 was used for Figure 2E by mistake. Below is the correct figure. The authors apologize for any inconvenience caused to the readers.

**Figure:**
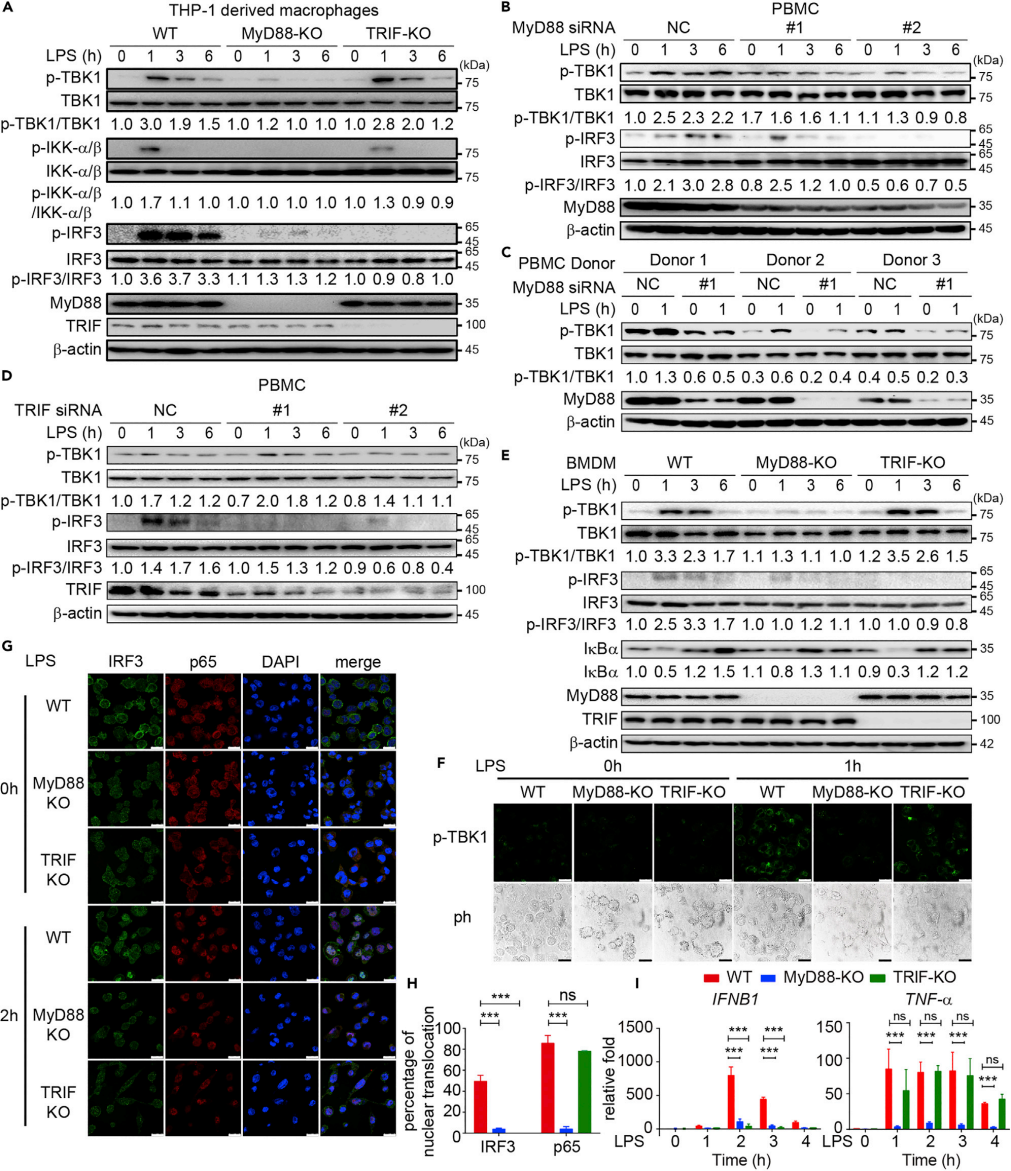
Figure 2. Both MyD88 and TRIF Are Critical for IRF3 Activation by LPS Treatment

