# Supplementary material for: Dual Feedforward Loops Modulate Type I Interferon Responses and Induce Selective Gene Expression during TLR4 Activation
Source: iScience. 2020 Feb 1;23(2):100881. doi: 10.1016/j.isci.2020.100881 (PMC7021547; doi:10.1016/j.isci.2020.100881)
Supplement: Document S1. Transparent Methods, Figures S1–S5, and Tables S1–S8 [file mmc1.pdf]

iScience, Volume *23*

# Supplemental Information

# Dual Feedforward Loops Modulate Type I Interferon Responses and Induce Selective Gene Expression during TLR4 Activation

**Jie Zhou, Tingzhe Sun, Shouheng Jin, Zhiyong Guo, and Jun Cui**

**Supplemental figures**

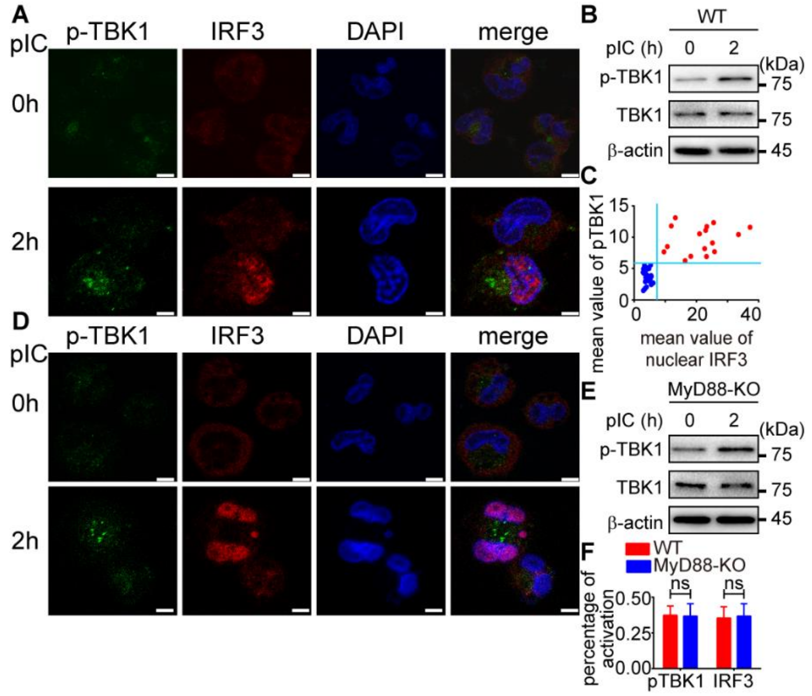


**Figure S1. TBK1 phosphorylation varies among cells under poly (I:C) stimulation, Related to Figure 1.**

(**A-B**) Wild type (WT) THP-1-derived macrophages were treated with poly (I:C) (pIC, 50 mg/mL) for 2 hours. After poly (I:C) stimulation, TBK1 phosphorylation and IRF3 nuclear translocation were imaged by con-focal microscope (**A**), and total cell lysates from treated cells were harvested and TBK1 phosphorylation was detected by immunoblotting (**B**). (**C**) The relative mean value of phosphorylated TBK1 and nuclear IRF3 in single cells (n=42) of (A) were measured by ImageJ. Cells with different level of TBK1 phosphorylation and nuclear translocated IRF3 were divided into two groups (blue group with lower level of TBK1 phosphorylation and IRF3 nuclear translocation while red group containing higher level of TBK1 phosphorylation and IRF3 nuclear translocation). (**D-E**) MyD88-KO THP-1-derived macrophages were treated with pIC (50 mg/mL) for 2 hours, and TBK1 phosphorylation and IRF3 nuclear translocation were imaged by con-focal microscope (**D**), while total cell lysates from treated cells were harvested and TBK1 phosphorylation was detected by immunoblotting (**E**). (**F**) Positive rate of TBK1 phosphorylation and IRF3 nuclear translocation under poly (I:C) stimulation for 2 hours of (**A**) and (**D**) were quantified by ImageJ. Data are representative of three independent experiments. At least 40 cells of (**A**) and (**D**) were analyzed for each group. The magnification is X1000 and the scale bar represents 10 μm. ns ($P > 0.05$) using student t-test.

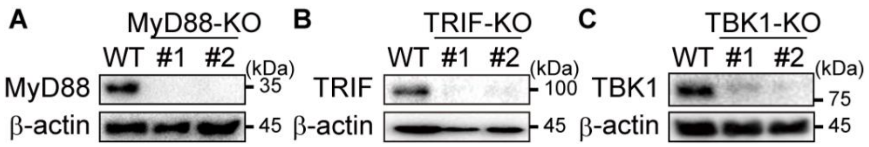


**Figure S2. Construction of THP-1 knocked out cell lines, Related to Figure 2.**

Immunoblot analysis of indicated proteins from MyD88 (**A**), TRIF (**B**) or TBK1 (**C**) knock-out (KO) THP-1 cells generated by CRISPR/Cas9 system.

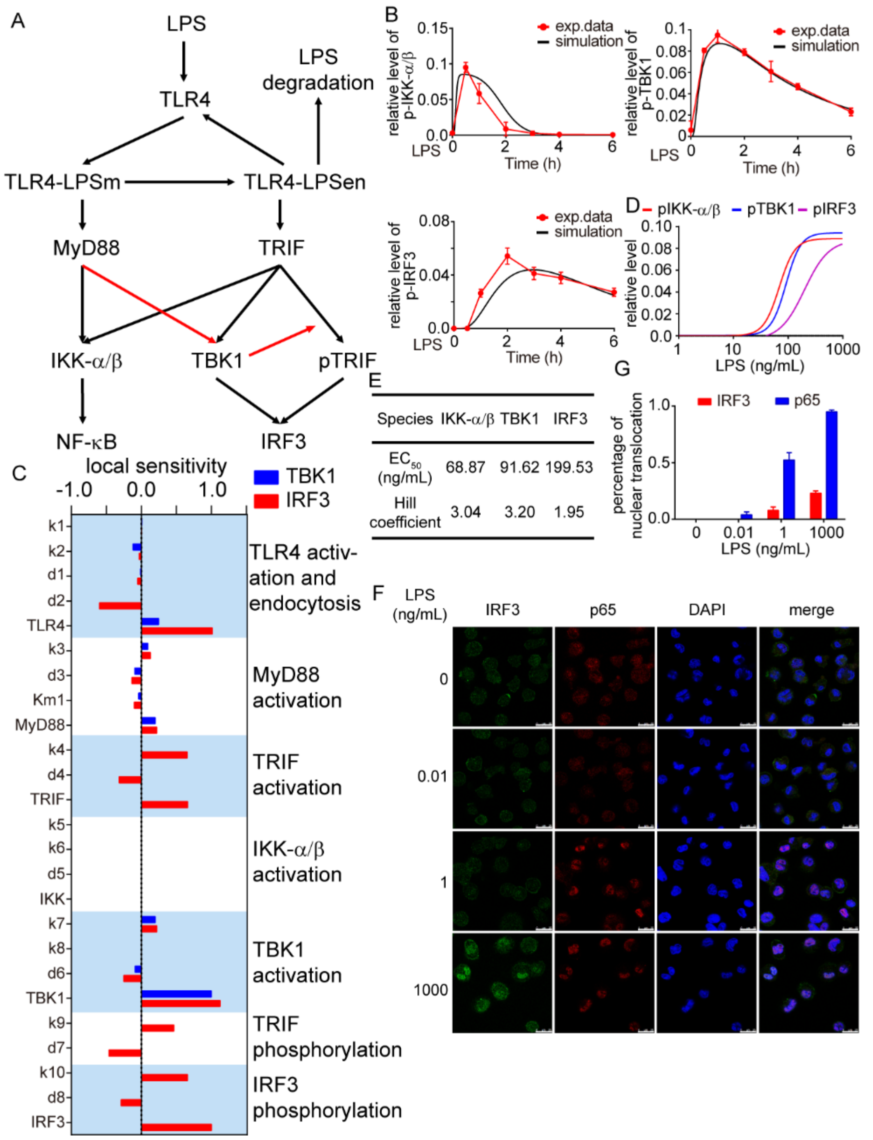


**Figure S3. Construction of mathematical model of TLR4 signaling pathway, See also Table S3-4. Related to Figure 4.**

(**A**) Schematic representation of mathematical model of TLR4 signaling pathway (**A**). (**B**) Model simulations (black solid lines) and experimental data (red dots linked with solid line) were measured in THP-1-derived macrophages stimulated with LPS (200 ng/mL). (**C**) Local sensitivity analysis of TBK1 and IRF3 phosphorylation with respect to kinetic parameters. The blue and red bars represent the sensitivity coefficients of TBK1 and IRF3 activation, respectively. (**D**) EC50 concentrations and Hill coefficients of indicated species under LPS treatment. (**E**) Simulation of maximal level of phosphorylated IKK-α/β, TBK1 and IRF3 under indicated concentration of LPS. (**F-G**) THP-1-derived macrophages were treated with indicated concentration of LPS for 2 hours, nuclear translocation of IRF3 and p65 were imaged by con-focal microscope (**F**) and quantified by ImageJ. 50 cells were analyzed for each group (**G**). The magnification is X400 and the scale bar represents 25μm. Data are representative of three independent biological experiments (shown as mean±SEM in B).

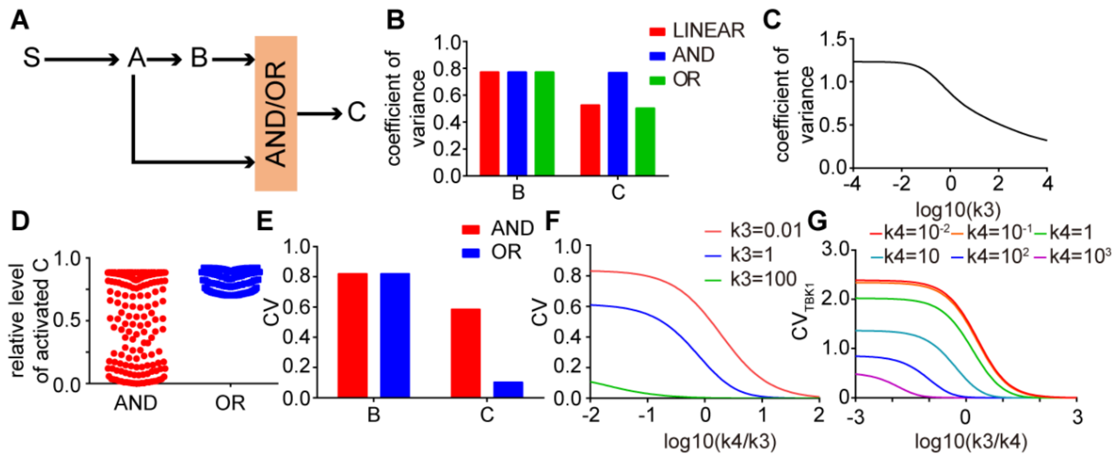


**Figure S4. Type I coherent feedforward loop shapes the heterogeneity of the activation of signaling molecules, See also Table S5-6. Related to Figure 4.**

(**A**) Model of the type I feedforward loop with AND logic or OR logic. (**B**) Coefficient of variation of B and C were compared when stochastic input was introduced into LINEAR model, C1-FFL with AND logic and C1-FFL with OR logic. (**C**) Within model containing C1-FFL with AND logic, the coefficient of variance of C activation was decreased while the value of $k_3$ was increased. (**D-E**) The relative level (**D**) and coefficient of variation (**E**) of C activation were compared when stochastic activation of B was introduced into C1-FFL with AND logic and C1-FFL with OR logic. (**F**) Within C1-FFL with OR logic, coefficient of variation of C were calculated when different value of $k_3$ and $k_4$ were chosen. (**G**) Within model containing both FFLs in (Figure 4D), effects of $CV_{TBK1}$ by parameters of TBK1 activation were simulated. Pairs of parameters of MyD88-induced TBK1 activation (k3) and TRIF-induced TBK1 activation (k4) were chosen and $CV_{TBK1}$ was calculated.

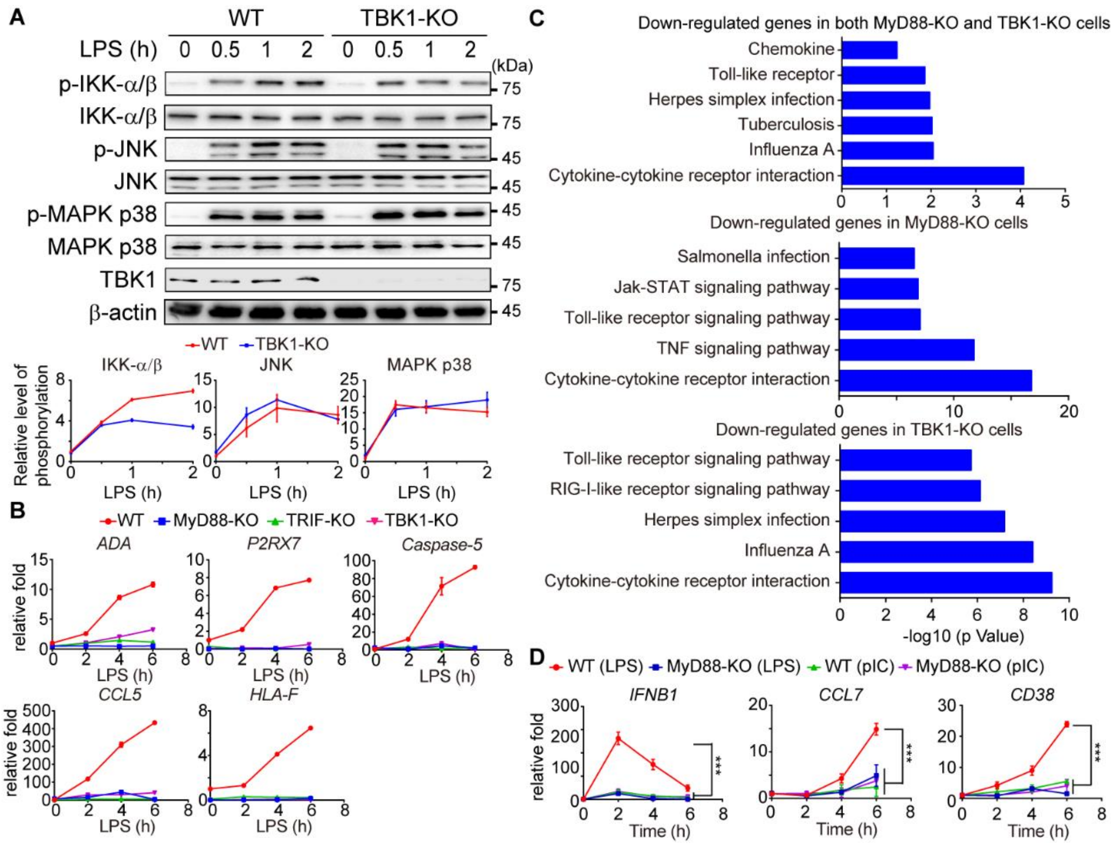


**Figure S5. Comparison of the expression of MyD88-dependent genes under LPS and poly (I:C) stimulation, Related to Figure 5.**

(**A**) Wild type (WT), or TBK1-knock-out (TBK1-KO) THP-1-derived macrophages were stimulated with LPS (200 ng/mL) for indicated time points. Phosphorylation of indicated proteins was detected by immunoblot (IB) analyses. Phosphorylation of IKK-α/β, JNK and MAPK p38 in three independent experiments was calculated and plotted. (**B**) Quantitative real-time PCR (qRT-PCR) analysis of indicated genes in WT and other indicated KO THP-1-derived macrophages was performed under LPS stimulation for 6 hours. (**C**) Gene ontology analysis for down-regulated genes in both MyD88-KO and TBK1-KO cells or in either KO cells. (**D**) qRT-PCR analysis of *IFNB1*, *CCL7* and *CD38* in wild type (WT) and MyD88-knockout (KO) THP-1-derived macrophages under LPS (200 ng/mL) or poly (I:C) (pIC, 50 μg/mL) stimulation for indicated time points. Data are representative of three independent biological experiments (shown as mean±SEM). ***$P < 0.001$ using two-way ANOVA test.

## Supplemental tables

**Table S1. Sequences of small guide RNA used in generation of HEK 293T and THP-1 knockout cell lines by CRISPR/Cas9, Related to Figure 2.**

| Gene | sgRNA sequence (5’-3’) |
|---|---|
| MyD88 #1 | GCTCCAGCAGCACGTCGTCG |
| MyD88 #2 | CTCGAGCAGTCGGCCTACAG |
| TRIF #1 | CCATTGACGGTGTTTCGGAC |
| TRIF #2 | ACGCCCCATTGACGGTGTTT |
| TBK1 #1 | TTTGAACATCCACTGGACGA |
| TBK1 #2 | CATAAGCTTCCTTCGTCCAG |

**Table S2. Sequences of primers used in quantitative real-time PCR analysis, Related to Figure 2.**

| Primer | Sequence (5'-3') |
| --- | --- |
| qp-RLP13A-F | GCCATCGTGGCTAAACAGGTA |
| qp-RLP13A-R | GTTGGTGTTCATCCGCTTGC |
| qp-IFNB1-F | CAGCAATTTTCAGTGTCAGAAGC |
| qp-IFNB1-R | TCATCCTGTCCTTGAGGCAGT |
| qp-TNF-α-F | CCAGACCAAGGTCAACCTCC |
| qp-TNF-α-R | CAGACTCGGCAAAGTCGAGA |
| qp-CCL7-F | AAAGCCTCTGCAGCACTTCT |
| qp-CCL7-R | GTGGCTACTGGTGGTCCTTC |
| qp-CD38-F | AAGTTGCCATTAGCTCCCCC |
| qp-CD38-R | GCAGCCACTTCAAGGGGTAT |
| qp-CXCL10-F | GTGGCATTCAAGGAGTACCTC |
| qp-CXCL10-R | TGATGGCCTTCGATTCTGGATT |
| qp-CXCL11-F | GACGCTGTCTTTGCATAGGC |
| qp-CXCL11-R | GGATTTAGGCATCGTTGTCCTTT |
| CD38ChIP-F | AGGTGGTTGACCAGGCATTT |
| CD38ChIP-R | TACTCCCTCCGCTAACTCCC |
| CXCL10ChIP-F | TTTGGAAAGTGAAACCTAATTCA |
| CXCL10ChIP-R | AAAACCTGCTGGCTGTTCCTG |

**Table S3. Equations of mathematical model of TLR4 signaling, Related to Figure 4.**

| **Reactions** | **Reaction rates** |
|---|---|
| **Ligand binding** | |
| TLR4 + LPS → TLR4-LPSm | k1*[TLR4]*[LPS] |
| TLR4-LPSm → TLR4 + LPS | d1*[TLR4-LPSm] |
| TLR4-LPSm → TLR4-LPSen | k2*[TLR4-LPSm] |
| TLR4-LPSen → TLR4 | d2*[TLR4-LPSen] |
| | |
| **Adaptor activation** | |
| TLR4-LPSm + MyD88 → TLR4-LPSm + aMyD88 | k3*[TLR4-LPSm]*[MyD88]^3/(Km1^3+[MyD88]^3) |
| aMyD88 → MyD88 | d3*[aMyD88] |
| TLR4-LPSen + TRIF → TLR4-LPSen + aTRIF | k4*[TLR4-LPSen]*[TRIF] |
| aTRIF → TRIF | d4*[aTRIF] |
| | |
| **Kinase activation** | |
| IKK-β + aMyD88 → pIKK-β + aMyD88 | k5*[aMyD88]*[IKK-β] |
| IKK-β + aTRIF → pIKK-β + aTRIF | k6*[aTRIF]*[IKK-β] |
| pIKK-β → IKK-β | d5*[pIKKβ] |
| TBK1 + aMyD88 → pTBK1 + aMyD88 | k7*[aMyD88]*[TBK1] |
| TBK1 + aTRIF → pTBK1 + aTRIF | k8*[aTRIF]*[TBK1] |
| pTBK1 → TBK1 | d6*[pTBK1] |
| | |
| **TRIF phosphorylation** | |
| TRIF + pTBK1 → pTRIF + pTBK1 | k9*[pTBK1]*[aTRIF] |
| pTRIF → aTRIF | d7*[pTRIF] |
| | |
| **IRF3 phosphorylation** | |
| IRF3 → pIRF3 | k10*[pTBK1]*[pTRIF]*[IRF3] |
| pIRF3 → IRF3 | d8*[pIRF3] |
| | |
| **Gene induction** | |
| pIRF3 → pIRF3 + *CD38* | k11*[pIRF3]/(Km2+[pIRF3]) |
| *CD38* → Ø | d9*[CD38] |
| pIRF3 → pIRF3 + *CXCL10* | k12*[pIRF3]/(Km3+[pIRF3]) |
| *CXCL10* → Ø | d10*[CXCL10] |

**Table S4. Description and values of parameters of mathematical model of TLR4 signaling, Related to Figure 4.**

| Symbols | Description | Values | Reference value | Source |
|---|---|---|---|---|
| k1 | Association rate of LPS and TLR4 at cell membrane | 5.59E+00 | 2.77E-02 | (Cheng et al. 2017) |
| k2 | Endocytosis rate of TLR4-LPSm | 8.86E-01 | 6.57E-02 | |
| k3 | Activation rate of MyD88 by TLR4-LPSm | 2.51E+02 | 1.50E+02 | |
| k4 | Activation rate of TRIF by TLR4-LPSen | 3.55E+01 | 1.80E+01 | |
| k5 | Activation rate of IKK-β by aMyD88 (activated MyD88) | 4.31E+02 | 5.50E+01 | |
| k6 | Activation rate of IKK-β by aTRIF (activated TRIF) | 1.88E-01 | 1.60E+00 | |
| k7 | Activation rate of TBK1 by aMyD88 | 5.56E+01 | - | |
| k8 | Activation rate of TBK1 by aTRIF | 6.95E+00 | 1.10E-02 | |
| k9 | Phosphorylation rate of aTRIF by pTBK1 | 8.71E+02 | - | |
| k10 | Phosphorylation rate of IRF3 by pTBK1 and pTRIF | 7.00E+03 | - | |
| k11 | Induction rate of *CD38* | 5.00E-01 | - | |
| k12 | Induction rate of *CXCL10* | 5.00E-01 | - | |
| d1 | Dissociation rate of TLR4-LPSm | 6.93E-02 | 2.77E-02 | (Cheng et al. 2017) |
| d2 | Degradation rate of LPS in endosome | 3.78E+00 | 2.00E+00 | |
| d3 | Inactivation rate of aMyD88 | 2.21E+01 | 2.20E+03 | |
| d4 | Inactivation rate of aTRIF | 1.36E+00 | 4.00E-02 | |
| d5 | Dephosphorylation rate of pIKK-β | 4.89E+00 | 9.00E-01 | (Werner et al. 2008) |
| d6 | Dephosphorylation rate of pTBK1 | 2.88E-01 | 3.58E-02 | (Cheng et al. 2017) |
| d7 | Dephosphorylation rate of pTRIF | 5.07E+01 | - | |
| d8 | Dephosphorylation rate of pIRF3 | 3.05E-01 | 1.01E-02 | (Cheng et al. 2017) |
| d9 | Degradation rate of *CD38* | 5.00E-01 | - | |
| d10 | Degradation rate of *CXCL10* | 5.00E-01 | - | |
| Km1 | EC50 of MyD88 activation | 4.28E-03 | 1.24E-02 | (Cheng et al. 2017) |

| | | | | |
|---|---|---|---|---|
| Km2 | EC50 of *CD38* induction | 1.00E-02 | - | |
| Km3 | EC50 of *CXCL10* induction | 1.00E-05 | - | |
| | | | | |
| TLR4 | Abundance of TLR4 | 2.00E-02 | 2.00E-02 | (Cheng et al. 2017) |
| MyD88 | Abundance of MyD88 | 1.00E-01 | 1.00E-01 | |
| TRIF | Abundance of TRIF | 1.00E-01 | 1.00E-01 | |
| IKK-β | Abundance of IKK-β | 1.00E-01 | 1.00E-01 | |
| TBK1 | Abundance of TBK1 | 1.00E-01 | 1.00E-01 | |
| IRF3 | Abundance of IRF3 | 1.00E-01 | 1.00E-01 | |

**Table S5. Equations of models for feedforward loops analysis, Related to Figure 4.**

| **Reactions** | **Reaction rates** |
| --- | --- |
| **LINEAR** | |
| S → Ø | d1*[S] |
| A + S → aA + S | k1*[S]*[A] |
| aA → A | d2*[aA] |
| aA + B → aA + aB | k2*[aA]*[B] |
| aB → B | d3*[aB] |
| aB + C → aB + aC | k3*[aB]*[C] |
| aC → C | d4*[aC] |
| | |
| **C1-FFL with OR logic** | |
| S → Ø | d1*[S] |
| A + S → aA + S | k1*[S]*[A] |
| aA → A | d2*[aA] |
| aA + B → aA + aB | k2*[aA]*[B] |
| aB → B | d3*[aB] |
| aB + C → aB + aC | k3*[aB]*[C] |
| aA + C → aA + aC | k4*[aA]*[C] |
| aC → C | d4*[aC] |
| | |
| **C1-FFL with AND logic** | |
| S → Ø | d1*[S] |
| A + S → aA + S | k1*[S]*[A] |
| aA → A | d2*[aA] |
| aA + B → aA + aB | k2*[aA]*[B] |
| aB → B | d3*[aB] |
| aA+ aB + C → aA + aB + aC | k3*[aA]*[aB]*[C] |
| aC → C | d4*[aC] |

**Table S6. Description and values of parameters of simplified models for feedforward loops analysis, Related to Figure 4.**

| Symbols | Description | Values | | |
|---|---|---|---|---|
| | | LINEAR | AND | OR |
| k1 | Activation rate of A by S | 1.00E+00 | 1.00E+00 | 1.00E+00 |
| k2 | Activation rate of B by activated A | 1.00E+00 | 1.00E+00 | 1.00E+00 |
| k3 | Activation rate of C by activated B/ A & B | 3.00E+00 | 3.00E+00 | 3.00E+00 |
| k4 | Activation rate of C by activated A | - | - | 3.00E+00 |
| d1 | Degradation rate of S | 1.00E+00 | 1.00E+00 | 1.00E+00 |
| d2 | Inactivation rate of activated A | 1.00E+00 | 1.00E+00 | 1.00E+00 |
| d3 | Inactivation rate of activated B | 1.00E+00 | 1.00E+00 | 1.00E+00 |
| d4 | Inactivation rate of activated C | 1.00E+00 | 1.00E+00 | 1.00E+00 |
| | | | | |
| A | Abundance of A | 3.00E+00 | 3.00E+00 | 3.00E+00 |
| B | Abundance of B | 3.00E+00 | 3.00E+00 | 3.00E+00 |
| C | Abundance of C | 3.00E+00 | 3.00E+00 | 3.00E+00 |

**Table S7. Equations of simplified models for feedforward loops analysis, Related to Figure 4.**

| Reactions | Reaction rates |
|---|---|
| **without FFLs** | |
| LPS → Ø | d1*[LPS] |
| LPS + TRIF → LPS + aTRIF | k2*[LPS]*[TRIF] |
| aTRIF → TRIF | d3*[aTRIF] |
| TBK1 + aTRIF → pTBK1 + aTRIF | k4*[aTRIF]*[TBK1] |
| pTBK1 → TBK1 | d4*[pTBK1] |
| IRF3 → pIRF3 | k5*[pTBK1]*[IRF3] |
| pIRF3 → IRF3 | d5*[pIRF3] |
| **with FFL-1** | |
| LPS → Ø | d1*[LPS] |
| LPS + MyD88 → LPS + aMyD88 | k1*[LPS]*[MyD88]^3/(Km1^3+[MyD88]^3) |
| aMyD88 → MyD88 | d2*[aMyD88] |
| LPS + TRIF → LPS + aTRIF | k2*[LPS]*[TRIF] |
| aTRIF → TRIF | d3*[aTRIF] |
| TBK1 + aMyD88 → pTBK1 + aMyD88 | k3*[aMyD88]*[TBK1] |
| TBK1 + aTRIF → pTBK1 + aTRIF | k4*[aTRIF]*[TBK1] |
| pTBK1 → TBK1 | d4*[pTBK1] |
| IRF3 → pIRF3 | k5*[pTBK1]*[IRF3] |
| pIRF3 → IRF3 | d5*[pIRF3] |
| **with FFL-2** | |
| LPS → Ø | d1*[LPS] |
| LPS + TRIF → LPS + aTRIF | k2*[LPS]*[TRIF] |
| aTRIF → TRIF | d3*[aTRIF] |
| TBK1 + aTRIF → pTBK1 + aTRIF | k4*[aTRIF]*[TBK1] |
| pTBK1 → TBK1 | d4*[pTBK1] |
| IRF3 → pIRF3 | k5*[pTBK1]*[pTRIF]*[IRF3] |
| pIRF3 → IRF3 | d5*[pIRF3] |
| **with both FFLs** | |
| LPS → Ø | d1*[LPS] |
| LPS + MyD88 → LPS + aMyD88 | k1*[LPS]*[MyD88]^3/(Km1^3+[MyD88]^3) |
| aMyD88 → MyD88 | d2*[aMyD88] |
| LPS + TRIF → LPS + aTRIF | k2*[LPS]*[TRIF] |
| aTRIF → TRIF | d3*[aTRIF] |
| TBK1 + aMyD88 → pTBK1 + aMyD88 | k3*[aMyD88]*[TBK1] |
| TBK1 + aTRIF → pTBK1 + aTRIF | k4*[aTRIF]*[TBK1] |
| pTBK1 → TBK1 | d4*[pTBK1] |
| IRF3 → pIRF3 | k5*[pTBK1]*[pTRIF]*[IRF3] |
| pIRF3 → IRF3 | d5*[pIRF3] |

**Table S8. Description and values of parameters of simplified models for feedforward loops analysis, Related to Figure 4.**

| Symbols | Description | Values | | | |
|---|---|---|---|---|---|
| | | without FFLs | FFL-1 only | FFL-2 only | with both FFLs |
| k1 | Activation rate of MyD88 by LPS | 4.10E+00 | 4.10E+00 | 4.10E+00 | 4.10E+00 |
| k2 | Activation rate of TRIF by LPS | 2.50E-01 | 2.50E-01 | 2.50E-01 | 2.50E-01 |
| k3 | Activation rate of TBK1 by aMyD88 | - | 5.56E+01 | - | 5.56E+01 |
| k4 | Activation rate of TBK1 by aTRIF | 4.00E+03 | 8.95E+00 | 4.00E+03 | 8.95E+00 |
| k5 | Phosphorylation rate of IRF3 by pTBK1 (and pTRIF in FFL-2) | 7.82E+00 | 7.82E+00 | 9.00E+03 | 9.00E+03 |
| d1 | Degradation rate of LPS in endosome | 4.05E+00 | 4.05E+00 | 4.05E+00 | 4.05E+00 |
| d2 | Inactivation rate of aMyD88 | 0.00E+00 | 3.10E+00 | 0.00E+00 | 3.10E+00 |
| d3 | Inactivation rate of aTRIF | 1.04E+00 | 1.04E+00 | 1.04E+00 | 1.04E+00 |
| d4 | Dephosphorylation rate of pTBK1 | 3.80E-01 | 2.58E-01 | 3.80E-01 | 2.58E-01 |
| d5 | Dephosphorylation rate of pIRF3 | 7.25E-01 | 7.25E-01 | 1.20E-01 | 1.20E-01 |
| Km1 | EC50 of MyD88 activation | 0.00E+00 | 4.28E-03 | 0.00E+00 | 4.28E-03 |
| | | | | | |
| TLR4 | Abundance of TLR4 | 2.00E-02 | 2.00E-02 | 2.00E-02 | 2.00E-02 |
| MyD88 | Abundance of MyD88 | 1.00E-01 | 1.00E-01 | 1.00E-01 | 1.00E-01 |
| TRIF | Abundance of TRIF | 1.00E-01 | 1.00E-01 | 1.00E-01 | 1.00E-01 |
| IKK-β | Abundance of IKK-β | 1.00E-01 | 1.00E-01 | 1.00E-01 | 1.00E-01 |
| TBK1 | Abundance of TBK1 | 1.00E-01 | 1.00E-01 | 1.00E-01 | 1.00E-01 |
| IRF3 | Abundance of IRF3 | 1.00E-01 | 1.00E-01 | 1.00E-01 | 1.00E-01 |

**Transparent Methods**

**Cell culture, plasmids and reagents**

HEK 293T cells were cultured in DMEM medium (Hyclone) with 10% FBS (Gibco) incubated in a 5% $CO_2$ chamber (Thermo Fisher Scientific). THP-1 cells were cultured in RPMI 1640 (Gibco) containing 10% FBS, which is also incubated in the 5% $CO_2$ chamber. LPS was purchased from Sigma (L4391). Before stimulation, THP-1 cells were seeded in 6-well plates with the density of $1\times10^6$ cells/mL for immunoblot and qRT-PCR or in 15 mm dish (NEST) with the density of $2.5\times10^5$ cells/mL for immunofluorescence staining and differentiated into macrophages (THP-1-deirived macrophages) by treating with 100 nM PMA (P8139, Sigma) for 16 hours. After PMA treatment, the medium was replaced by fresh RPMI 1640 medium for 48 hours, and then cells were used for experiments. Blood from healthy donors (Zhongshan School of Medicine) was used for the isolation of PBMCs by ficoll-hypaque density-gradient centrifugation. The use of PBMCs was in compliance with institutional guidelines and approved protocols of Sun Yat-sen University. Bone marrow derived macrophages (BMDMs) were were cultured in RPMI 1640 (Gibco) containing 10% FBS and derived from bone marrow of 18–20 g C57BL/6 mice (Guangdong Medical Laboratory Animal Center) and cultured for 6–8 d with 100 ng/ml macrophage colony-stimulating factor (PeproTech). All of the following plasmids were generated with empty pcDNA3.1 vector, including Flag-TBK1, HA-TBK1, myc-MyD88, Flag-MyD88, Flag-MyD88 DD, Flag-MyD88 TIR.

**RNAi**

hMyD88-siRNA, hTRIF-siRNA and control (scramble) siRNA were obtained from Shanghai TranSheep Bio Co. Ltd. and transfected into HEK293T cells, pMs, and BMMs with RNAiMAX (Invitrogen) according to the manufacturer's instructions. siRNA sequences were as follows: scramble-siRNA: forward (5′-UUCUCCGAACGUGUCACGUTT-3′) and reverse (5′-ACGUGACACGUUCGGAGAATT-3′); hMyD88-siRNA 1#, forward (5′-GCACCUGUGUCUGGUCUAUTT-3′) and reverse (5′- AUAGACCAGACACAGGUGCTT-3′); hMyD88-siRNA 2#, forward (5′- CCCAUCAGAAGCGACUGAUTT-3′) and reverse (5′-AAUUUCUGUUCCGAUGAUGTT-3′); and hMyD88 -siRNA 3#, forward (5′-GCAUCCUGAGGUUCAUCACTT-3′) and reverse (5′- GUGAUGAACCUCAGGAUGCTT-3′). hTRIF-siRNA 1#, forward (5′-CAGCCUACCUCCAGAGCUATT-3′) and reverse (5′-UAGCUCUGGAGGUAGGCUGTT-3′); hTRIF-siRNA 2#, forward (5′-CAGCCUACCUCCAGAGCUATT-3′) and reverse (5′- AUCAGUCGCUUCUGAUGGGTT-3′); and hTRIF -siRNA 3#, forward (5′-CGAAAGGCCAUGUGGAGGATT-3′) and reverse (5′-UCCUCCACAUGGCCUUUCGTT-3′).

**Generation of HEK 293T and THP-1 knockout cell lines by CRISPR/Cas9**

HEK 293T and THP-1 knock-out cell lines were generated using lentiviral vector encoding Cas9 and certain small guide RNAs (sgRNA). The sgRNAs for certain gene were shown in Table S1.

**Luciferase reporter assays**

HEK 293T cells were planted in 24-well plates and transfected with plasmids expressing the ISRE luciferase reporter (firefly luciferase; 20 ng/well) and pRL-TK (Renilla luciferase plasmid;

8 ng/well) together with different plasmids as indicated. 24 hours after transfection, enzyme activity was detected and normalized by the efficiency of transfection compared to Renilla luciferase activity levels. Fold induction relative to basal level was measured in cells containing different plasmids. The values were means ± SEM of three independent transfections performed in parallel.

**Immunoprecipitation, immunoblot, native gel electrophoresis and antibodies**

Whole cell lysate was obtained with low-salt lysis buffer (50 mM Hepes (pH 7.5), 150 mM NaCl, 1 mM EDTA, 1.5 mM MgCl2, 10% glycerol, 1% Triton X-100), supplemented with protease inhibitor cocktail (5 mg/mL; Roche) after LPS stimulation for indicated time points. Protein samples were mixed with the 5×loading buffer (Cell Signaling Technology) and heated for 100°C for 5 min. For immunoblot assay, Mixture was resolved by SDS-PAGE. For immunoprecipitation, samples were incubated with anti-Flag agarose gels (Sigma) or protein A/G beads (Thermo Fisher Scientific) together with indicated antibody overnight. Beads were washed three to five times with low-salt lysis buffer, and immunoprecipitates were eluted with 3×SDS loading buffer (Cell Signaling Technology) and resolved by SDS-PAGE. For native gel electrophoresis, samples were subjected to electrophoresis using non-denaturing gel which contained 7.5% w/v polyacrylamide (acrylamide:bisacrylamide, 30:1). After electrophoresis, protein was transferred to polyvinylidene fluoride membranes (Bio-Rad Laboratories) and then blocked with 5% skim milk (BD) for 1 hour and incubated with appropriate antibody. LumiGlo Chemiluminescent Substrate System (KPL) was used to detect specific band of certain protein. Antibodies used in immunoblot can be listed as follows: Anti-IRF3 rabbit polyclonal antibody, anti-IRF3 mouse polyclonal antibody, goat anti–mouse IgG-HRP and goat anti–rabbit IgG-HRP antibodies were purchased from Santa Cruz Biotechnology Inc. Anti-phospho-IRF3 (Ser396) rabbit monoclonal antibody, anti-TBK1/NAK (D1B4) rabbit monoclonal antibody, anti-phospho-TBK1/NAK (Ser172) rabbit monoclonal antibody, anti-phospho-IKKα/β (Ser176/180) rabbit monoclonal antibody, IκBα mouse monoclonal antibody, anti-MyD88 rabbit monoclonal antibody, anti-TRIF rabbit polyclonal antibody, anti-NF-κB p65 (L8F6) mouse monoclonal antibody were purchased from Cell Signaling Technology. Anti-beta actin mouse monoclonal antibody was purchased from Proteintech. Anti-IKKα mouse antibody was purchased from Novus Biologicals. Anti-IKKβ mouse antibody was purchased from Millipore.

**Chromatin immunoprecipitation (ChIP)**

Approximately $2*10^7$ THP-1 derived macrophages were used for each ChIP, and cells were stimulated with IFN-β for 4 hours before collection. ChIP was performed using a protocol described in the website of ROCKLAND™ (*https://rockland-inc.com/Chromatin-Immunoprecipitation-Protocol.aspx*). Cells were cross-linked on plates with 1% formaldehyde for 30 minutes at room temperature, which was quenched with glycine. Cells were then collected, subjected to nuclear extraction and sonicated to fragment the DNA. Cell lysates were pre-cleared with protein A/G beads (Thermo Fisher Scientific) and incubated with protein A/G beads together with Anti-IRF3 rabbit polyclonal antibody (Santa Cruz Biotechnology Inc) or non-immune rabbit IgG overnight. After immunoprecipitation, immune complex was washed and eluted as protocol described. Cross-linking was reverted in high

salt buffer at 65℃ for 5 hours. RNase A and protease K were used to purify DNA. DNA was finally purified by phenol-chloroform and chloroform extractions. Samples were analyzed by qPCR using primers specifically recognized promoters of *CD38* and *CXCL10*. The enrichment was normalized to the one observed in non-immune IgG ChIP controls.

**RNA extraction, RNA sequencing and qRT-PCR analysis**

Total RNA was extracted from cells with TRIzol reagent (Life Technologies), according to the manufacturer's instructions. RNA extracts were sequenced on an Illumina HiSeq platform by HONOR TECH. 1μg RNA was used to obtain cDNA through reverse transcription with HiScript® II Q RT SuperMix for qPCR (+gDNA wiper) kit (Vazyme). qRT-PCR was performed using Lightcycler 480 SYBR green I Master (Roche) with 2x superStar PCR Mix (GeneStar). The primers used in qRT-PCR were listed in Table S2.

**Immunofluorescence staining, imaging and image analysis**

THP-1-derived macrophages were seeded and stimulated as above. After stimulation, cells were fixed, permeated and blocked as protocol, and then incubated with 1:150 indicated primary antibody overnight, followed by incubation with 1:750 secondary antibody for 1 hour. Phosphate buffer (PBS) was used to wash three times between each step. After staining, Leica TCS-SP5 confocal fluorescence microscope was used to acquire images.

**Mendeley Data**

Our data of RNA sequencing was submitted to the Sequence Read Archive (SRA) database and the accession number is SUB6564036.

**Statistical Analysis**

Data are represented as mean ± SEM when indicated. For analysis of significance, student t-test or two-way ANOVA followed with multiple comparisons was used. Differences between groups were considered significant when $P < 0.05$.

**The mathematical modeling of chimeric FFL of TLR4 signaling pathway**

We developed a chimeric FFL model with deterministic ordinary differential equations (ODEs), describing the processes of LPS-TLR4 binding, activation of MyD88 and TRIF, phosphorylation of IKK-β and TBK1, phosphorylation of TRIF and IRF3 (Figure 3A). The reactions within TLR4 signaling pathway were listed, refer to the TLR4 model of Cheng et al with proper simplification (Cheng et al., 2015) (Table. S3). It was reported that TLR4 recognized LPS on the cell membrane that activated MyD88, followed by endocytosis which activated TRIF. According to the parameters of published models, we fixed our parameters of LPS binding and adaptors activation. Cheng et al reported that activated MyD88 formed Myddosome which was inherently cooperative (Cheng et al., 2015), leading us to simulate this process using Hill kinetics while Hill coefficient equals 3. The phosphorylation of IKK-β, TBK1, TRIF and IRF3 was formalized by ODEs based on mass action kinetics. Since the threshold of IRF3 activation for MyD88-dependent downstream genes is higher than that of MyD88-independent downstream genes, induction of *CD38* and *CXCL10* was formalized by Michaelis-Menten equation using different value of $K_m$. The model was numerically integrated

in MATLAB R2016a (MathWorks). We first referred to the parameters of previously reported TLR4 models (Cheng et al., 2015; Kearns et al., 2006; Werner et al., 2005) and trained our parameters using pattern search algorithm with quantified data of phosphorylated IKK-β, TBK1 and IRF3 (Figure S3C). The final parameter values were shown in Table S4. The local sensitivity coefficients of kinetic parameters were calculated to quantitatively evaluate critical parameters and components in the signaling pathways (Wu et al., 2008) as follows:

$$S_{P_i}^{M} = \frac{P_i}{M} * \frac{dM}{dP_i} \quad (1)$$

in which M represents the peak amplitude of $P_i$, $P_i$ ($P_1$, $P_2$ . . . $P_n$) is the nonzero variable and kinetic parameters vector, and $P_i$ is the *i*th nonzero variable and kinetic parameter. The local sensitivity was calculated as the change in M aroused by a 1% change in each nonzero variable and kinetic parameter.

In Figure 4, we considered the different behavior of heterogeneity transduction by different FFLs that directly controlling activation of TBK1 and IRF3. For simplification, we first used a LINEAR model or model containing one FFL with AND or OR logic (Table S5-6). In order to investigate the transduction of heterogeneity, we provided a stochastic input (extrinsic noise, $\mu$ = 0 and $\sigma$ =1.5 for each log-normal distribution) and simulated the results and calculated the coefficient of variation (CV) using the formula:

$$CV_i = \frac{std\ (\mathrm{P}_{i,j})}{mean\ (\mathrm{P}_{i,j})} \quad (2)$$

(for i denotes TBK1 or IRF3, and j = 1, 2 … 200 representing the number of simulated cells) We then compared the heterogeneity transduction of FFL with different logic when activation of B was stochastic (extrinsic noise, $\mu$ = 0 and $\sigma$ =1.5 for each log-normal distribution). And we simulated the results and calculated the CV using the formula (2) with different parameter sets in Figure S4D-F. Next, we used a simplified model in which the details of TLR4-LPS binding module and adaptors activation module were omitted (Table. S7-8). Since stochastic activation of TRIF that raised by endocytosis (Cheng et al., 2015), we added cell-to-cell variability (extrinsic noise, $\mu$ = 0 and $\sigma = 1.5$ for each log-normal distribution) to TRIF activation, simulated activation of TBK1 and IRF3 in 200 single cells and calculated the CV of each model using the formula (2).

## Supplemental References

Kearns, J.D., Basak, S., Werner, S.L., Huang, C.S., and Hoffmann, A. (2006). IkappaBepsilon provides negative feedback to control NF-kappaB oscillations, signaling dynamics, and inflammatory gene expression. J Cell Biol *173*, 659-664.

Werner, S.L., Barken, D., and Hoffmann, A. (2005). Stimulus specificity of gene expression programs determined by temporal control of IKK activity. Science *309*, 1857-1861.

Wu, W.H., Wang, F.S., and Chang, M.S. (2008). Dynamic sensitivity analysis of biological systems. BMC Bioinformatics *9 Suppl 12*, S17.
